# Cone Genesis Tracing by the *Chrnb4*-EGFP Mouse Line: Evidences of Cellular Material Fusion after Cone Precursor Transplantation

**DOI:** 10.1016/j.ymthe.2016.12.015

**Published:** 2017-01-28

**Authors:** Sarah Decembrini, Catherine Martin, Florian Sennlaub, Sylvain Chemtob, Martin Biel, Marijana Samardzija, Alexandre Moulin, Francine Behar-Cohen, Yvan Arsenijevic

**Affiliations:** 1 Unit of Retinal Degeneration and Regeneration, Department of Ophthalmology, University of Lausanne, Hôpital ophtalmique Jules-Gonin, Fondation asile des aveugles, 1004 Lausanne, Switzerland; 2 Sorbonne Universités, UPMC/Univ Paris 06, UMRS 968, INSERM, U968, Institut de la Vision, 75012 Paris, France; 3 Departments of Pediatrics, Ophthalmology and Pharmacology, Hôpital Ste. Justine Research Center, Montreal, QC H3T1C5, Canada; 4 Center for Integrated Protein Science Munich CIPSM, Department of Pharmacy-Center for Drug Research, Ludwig-Maximilians-Universität München, 81377 München, Germany; 5 Laboratory for Retinal Cell Biology, Department of Ophthalmology, University of Zurich, 8952 Schlieren, Switzerland; 6 Pathology Laboratory, Department of Ophthalmology, University of Lausanne, Hôpital ophtalmique Jules-Gonin, Fondation asile des aveugles, 1004 Lausanne, Switzerland; 7 Department of Ophthalmology, University of Lausanne, Hôpital ophtalmique Jules-Gonin, Fondation asile des aveugles, 1004 Lausanne, Switzerland

**Keywords:** retina, transplantation, neurodegeneration, fusion, cones, retinal dystrophy

## Abstract

The cone function is essential to mediate high visual acuity, color vision, and daylight vision. Inherited cone dystrophies and age-related macular degeneration affect a substantial percentage of the world population. To identify and isolate the most competent cells for transplantation and integration into the retina, cone tracing during development would be an important added value. To that aim, the *Chrnb4*-EGFP mouse line was characterized throughout retinogenesis. It revealed a sub-population of early retinal progenitors expressing the reporter gene that is progressively restricted to mature cones during retina development. The presence of the native CHRNB4 protein was confirmed in EGFP-positive cells, and it presents a similar pattern in the human retina. Sub-retinal transplantations of distinct subpopulations of *Chrnb4*-EGFP-expressing cells revealed the embryonic day 15.5 high-EGFP population the most efficient cells to interact with host retinas to provoke the appearance of EGFP-positive cones in the photoreceptor layer. Importantly, transplantations into the DsRed retinas revealed material exchanges between donor and host retinas, as >80% of transplanted EGFP-positive cones also were DsRed positive. Whether this cell material fusion is of significant therapeutic advantage requires further thorough investigations. The *Chrnb4*-EGFP mouse line definitely opens new research perspectives in cone genesis and retina repair.

## Teaser

In adult mouse and human retina, the beta-4 subunit of the Acetylcholine receptor (CHRNB4) is expressed only in cones. The *Chrnb4*-EGFP mouse served for cone lineage-tracing and transplantation studies. Transplantation of *Chrnb4*-EGFP cone precursors into DsRed retina revealed frequent bidirectional cellular material exchanges between donor and host cones.

## Introduction

Human vision relies mainly on cone photoreceptors that mediate color, daylight, as well as sharp and central vision. Diseases resulting in cone photoreceptor loss, such as age-related macular degeneration (AMD), Stargardt disease, and other cone-rod dystrophies, lead to severe visual impairment of the day central vision or to legal blindness.^1^ Additionally, in Retinitis pigmentosa, although the disease affects the rod function and survival, cones degenerate secondarily to rod loss.^2^, ^3^, ^4^ While promising treatments based on deep analyses of the cone metabolism during degeneration^5^, ^6^ are currently in development, the pursuit of studies to reveal the molecular events driving cone genesis and degeneration is still necessary. Indeed in diseases where gene mutations affect only the rod function, different patterns of cone loss succeed rod death with, in certain cases, long-term preservation of macula cones,^7^ whereas other diseases lead to rapid cone death.^1^ The tracing and analyses of cones during development in animal models of these diseases will help to understand how cones are affected by rod dysfunction and to dissect death pathways.^1^

Many mechanisms of cone genesis remain elusive because of the difficulty to track the early cone generation processes. Nonetheless, important cone development features have already been identified. Rod and cone photoreceptors derive from retinal progenitor cells that express the bHLH transcription factor OLIG2 and the homeobox transcription factor orthodenticle homeobox 2 (OTX2).^8^ The photoreceptor generation is coordinated by the transcription factors OTX2 and cone rod homeobox (CRX).^9^, ^10^ The presence of OTX2 and transiently Onecut1 (OC1) induces the cone differentiation program.^11^ Newborn cone cells turn on OTX2, the retinoic acid receptor (RAR), and other retinoids X and orphan receptor families, such as retinoid X receptor gamma protein (RXRγ) and ROR, during cone maturation.^12^ Unfortunately, only a few mouse lines expressing reporter genes during the early cone development were described to better study gene networking.^13^, ^14^, ^15^, ^16^, ^17^, ^18^

A recent paper described the tracing of cones and bipolar cells in a transgenic mouse with Coiled-Coil Domain Containing 136 (Ccdc136) GFP-trapped allele.^19^ To investigate the integration capacity of cones during their formation, embryonic day (E)17.5 cells were transplanted into the retina of wild-type mice. Several EGFP-positive cells were detected in the recipient retina with apical and basal processes, but they did not express cone markers.

To follow developing neurons, the gene expression nervous system atlas (GENSAT) project recently provided hundreds of mouse reporter lines expressing the EGFP reporter gene under the control of different bacterial artificial chromosomes (BACs) (http://www.gensat.org). About 100 genes were found to be restricted to a stratum or cell type of the retina (http://www.gensat.org/retina.jsp).^20^, ^21^ Among these, the mouse line *Chrnb4*-EGFP specifically expresses EGFP in adult cones.^20^ CHRNB4 is the beta-4 subunit of the Acetylcholine receptor^22^ not yet characterized during retinal embryogenesis. In this work, we describe the expression of the *Chrnb4*-EGFP reporter line and correlate it with the presence of the endogenous CHRNB4 protein during cone genesis. We show that CHRNB4 expression is detected in cones since their early genesis, before the appearance of OTX2. Indeed, between E12 and E14, the EGFP expression was preferentially found in proliferating retinal progenitors and then in cones positive for RXRγ. After E15, the EGFP expression is progressively restricted to cone precursor cells. A similar expression pattern also was observed in the fetal and adult human retinas.

Transplantation studies confirmed these immunohistological observations and revealed that only E15.5 high-EGFP cells interact with the host retina. Yet, it should be emphasized that >80% of the EGF-positive cells detected in the outer nuclear layer (ONL) of the recipient retinas are the result of cell material exchanges between the graft and the host cells. The mechanism of cell material fusion remains to be determined, and it merits a deep investigation to evaluate the possibility of using this phenomenon as a therapeutic approach to restore a cone function in models of cone dystrophies.

The *Chrnb4*-EGFP mouse line thus allows the tracing and isolation of cones during different stages of retinogenesis, opening new research perspectives on cone genesis, physiology, and pathology.

## Results

### Validation of *Chrnb4*-EGFP Expression during the Retina Development: Evidence of CHRNB4 Expression in a Subpopulation of Developing Photoreceptors

As previously highlighted in the Introduction, the *Chrnb4*-EGFP transgenic mouse line expresses EGFP in adult cones.^20^ In the present work, we first investigated when CHRNB4 is activated in the embryonic retina and if the expression can help to track the early stage of cone genesis. The expression pattern of the *Chrnb4*-EGFP transgene during retinogenesis was assessed by immunohistochemistry against the reporter gene EGFP (to enhance the detection of the signal). In the following text, we use the term progenitors for cells with a high potential and a large capacity to proliferate and precursors for cells that are already specified, for instance, neuroblasts (as discussed in Cayouette et al.^23^ and Merkle and Alvarez-Buylla^24^).

Retinal cells originate from two partially overlapping waves of generation: first from E10 to E20 and second from E14 to post-natal day (P)10. In rodents, blue cones are born early, starting from E10, together with other early-born neurons, the ganglion, and horizontal cells.^25^, ^26^ The different amacrine cells are generated between E10 and P6.^26^, ^27^ The last red and green cones are born from E16 on. Rod photoreceptors are born between E14 and P7, thus, the period of rod genesis overlaps that of the last cones.^27^ Cones and rods finalize their differentiation in the apical part of the retina.

Between E12 and E14, the S-phase nuclei of retinal progenitors also are localized at the basal retinal side. Moreover, the nuclei of proliferating retinal progenitors migrate throughout the retina.

**Figure 1:**
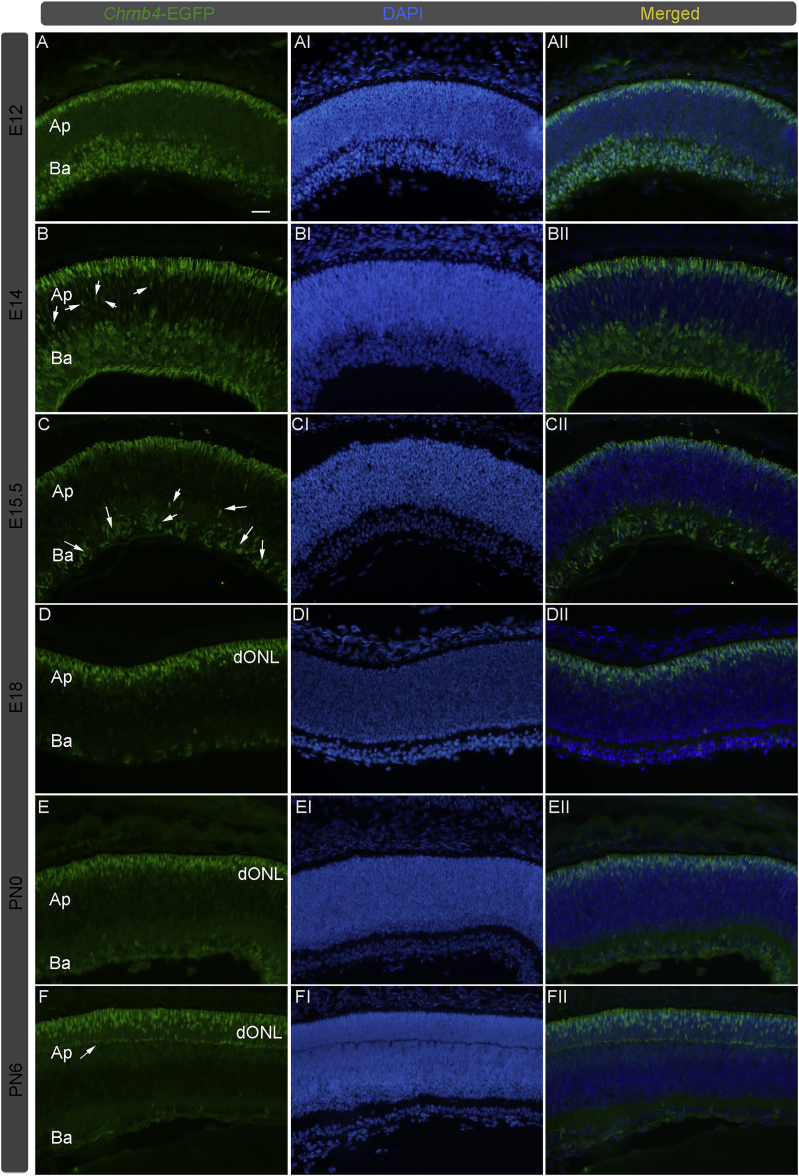
Progressive Restriction of *Chrnb4*-EGFP Expression in Developing Photoreceptors throughout Retinogenesis (A–FII) Transversal sections of retinas at different developmental stages were collected from *Chrnb4-*EGFP reporter mice. (A–BII) EGFP detection is shown in round and elongated cells adjacent to the basal and to the most apical retinal region in E12 and E14 retinas. (B) White arrows indicate spindled cells in the central part of the retina expressing weak EGFP (probably progenitors). (C–CII) The EGFP expression is more restricted at the edge of the retinal apical side of E15.5 retinas. Few round cells at the basal retinal side still show EGFP expression (white arrows). (D–EII) In E18 and PN0 retinas, EGFP expression is exclusively restricted to the developing outer nuclear layer (dONL) in which photoreceptors differentiate. (F–FII) EGFP is expressed in a subset of photoreceptor cells at PN6, not in all photoreceptors. (F) The white arrow indicates the margin of the dONL. DAPI, nuclear staining (blue); Ba, basal; Ap, apical; PN, post-natal day; E, embryonic day. Scale bars, 20 μm.

The EGFP expression was detected for the first time in most cells belonging to the developing optic cup at E11, where retinal progenitors are actively proliferating (data not shown). From E12 to E14, the high-EGFP intensity was localized in round and in a few elongated cells adjacent to the basal and to the most apical retinal regions, respectively (Figures 1A–1BII). This latter region corresponds to developing photoreceptors. In addition, many spindled cells expressing a less intense EGFP signal were observed between the apical and basal sides of the retina, where progenitors undergo cell division (Figures 1B–1BII, retinal progenitors indicated with white arrows). As different retinal cell types might be localized at the basal side of the retina, a further in-depth characterization is required to better identify the EGFP-positive cells. However, in this study we focused on the cone generation only.

Then, between E15.5 and E18, the EGFP expression pattern becomes progressively restricted to the most apical side of the developing retina, where cone precursors undergo their terminal differentiation and several late-stage progenitors proliferate. At this stage, only rare EGFP-positive cells were still detected in the middle retina, where progenitors are active (Figures 1C–1DII). These results suggest that the EGFP expression is present in a subpopulation of early retinal progenitors, and it is then progressively restricted to early-born photoreceptors in this apical region.

In post-natal retinas, the EGFP signal was further limited to cells localized more apically in the developing ONL, in which both types of photoreceptors, rods and cones, are confined (Figures 1E–1EII). From P6, the small number of EGFP-positive photoreceptors localized in the developing ONL (Figure 1F, white arrows denote ONL margin) suggests that the EGFP expression is limited to a subpopulation of photoreceptor cells, probably cones (Figures 1F–1FII, see below). At this age, almost all the EGFP-positive cells located in the ONL express the GNAT2 cone marker, and none shows the rod markers GNAT1 or Rhodopsin (Figure S1). Gene expression analysis of the EGFP-positive cells compared to negative cells confirmed the cone phenotype with other markers (Figures S1A–S1AI). Interestingly, the *Rhodopsin* mRNA is present in these cells (at a lower level compared to cone-specific mRNAs), but the protein is not detected by immunohistochemistry.

Co-staining with antibodies against the EGFP and CHRNB4 endogenous protein, as shown in Figures S2A–S2CII and summarized in the related histogram (Figure S2D), revealed that, in E12.5, E15.5, and adult retinas, most of the EGFP-positive cells co-expressed the CHRNB4 protein (85.3% ± 4.5%, 94.3% ± 2.1%, and 99% ± 0.9%, respectively) at the cell periphery. Notably, all the CHRNB4-positive cells were EGFP positive (data not shown). The lower percentage of EGFP-positive cells co-labeled with CHRNB4 at E12.5 may be ascribable to the potentially weaker signal intensity of CHRNB4 native proteins at this developmental stage.

### The Peak of Birth of Early EGFP-Positive Cells Corresponds to the Cone Generation Wave

The above results suggest the progressive restriction of the EGFP expression over time in developing cones. To investigate whether *Chrnb4*-EGFP is expressed in early retinal progenitors, a cell proliferation assay was performed to distinguish early from later retinal progenitors. The retinal progenitors are not limited to producing one single type of retinal neuron, but the frequency of generating specific fates changes over time.^28^ The majority of cones become post-mitotic before E16 and rods start being generated meanwhile.

**Figure 2:**
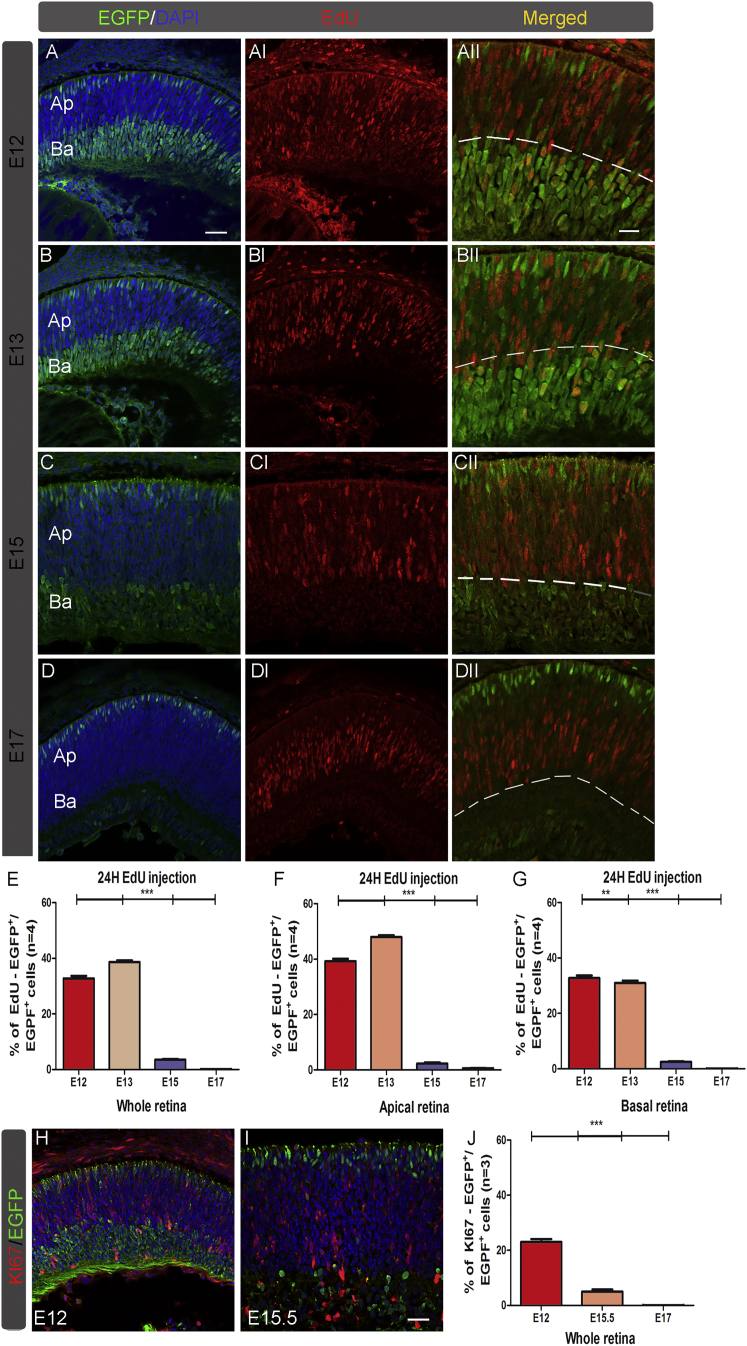
Investigation of the Chrnb4-EGFP-Positive Cell Proliferation during the Cone Generation Wave Pregnant females were labeled for 24 hr with EdU at E12, E13, E15, or E17. (A–DII) Transversal sections are shown of retinas at E13, E14, E16, and E18 stained for EGPF and EdU. (A–D) Merged pictures show EGFP (green) and DAPI nuclear staining (in blue). (AI–DI) EdU labelings (red) at different developmental stages are shown. (AII–DII) Merged pictures show EdU-positive cells (red) and EGFP-positive cells (in green). Dashed lines delimit the apical (Ap) from the basal (Ba) retinal regions used for counting in (F) and (G). (E–G) Percentage of double EdU-/EGFP -positive cells were counted over the EGFP-positive cells detected in the whole (E), apical (F), and basal (G) retina. (H–J) Percentage of double Ki67- and EGFP-positive cells counted over the total number of EGFP-positive cells confirms the results achieved with the EdU labeling. DAPI, nuclear staining (blue); Ba, basal; Ap, apical. (E–G and J) ANOVA with Tukey’s correction, ***p < 0.001; n = 4. Error bars correspond to SEM. Scale bars, 25 μm (A–D, H, AI–DI, and HI) and 15 μm (AII–DII and HII).

To this end, pregnant females were pulse-labeled with 5-ethynyl-2′-deoxyuridine (EdU), a thymidine analog, for 24 hr at gestation days 12, 13, 15, and 17. After 24 hr, embryonic retinas were collected and analyzed for the EGFP and EdU co-expressions (Figures 2A–2G). As summarized in Figures 2A–2DII and quantified in the corresponding histogram (Figure 2E), the percentage of the total EGFP-positive cells incorporating EdU rapidly decreased between E13 (36.1% ± 1.9%) and E15 (3.4% ± 0.5%), underlining the fact that the large majority of EGFP-positive cells became post-mitotic between E14 and E15, when rod photoreceptors start to differentiate. Then, the apical (photoreceptor precursors and progenitors) and the basal retinal regions (post-mitotic ganglion and amacrine cells as well as progenitors from the first-generation wave) were separately analyzed for EdU incorporation (Figures 2A–2G). To focus on cone development, the apical retinal side was investigated. The highest number of EGFP-positive cells incorporating EdU was observed at E12 and E13. Then a rapid drop of this cell population occurred between these ages and E15, when only 2.3% ± 0.3% of the EGFP-positive cells proliferated and were post-mitotic afterward (Figure 2F). Considering that cones are the most abundant cell type generated between E14 and E15, that after E15 rare proliferating cells were EGFP positive, and that only a few retinal progenitors just start giving rise to rods at this age,^26^ we hypothesized that apical EGFP-positive cells might be cone precursors.

In the basal region, a robust EdU incorporation in *Chrnb4*-EGFP cells occurred similarly between E12 and E14, and it was then dramatically reduced at E15 (Figure 2G). Overall, Ki67 staining confirmed this pattern of cell proliferation (Figures 2H–2J).

These results reveal the competence of the *Chrnb4*-EGFP line to trace early progenitors and early newborn neurons, but not later-born cells, such as rods, bipolar cells, or Müller glia cells. Moreover, the present data indicate a transient EGFP expression in basally localized cells, necessitating a deeper analysis to identify in which cell population CHRNB4 is expressed.

### During Early Retinal Development, *Chrnb4*-EGFP Is Expressed in Cells Containing Essential Factors for Cone Development, such as OTX2 and RXRγ

**Figure 3:**
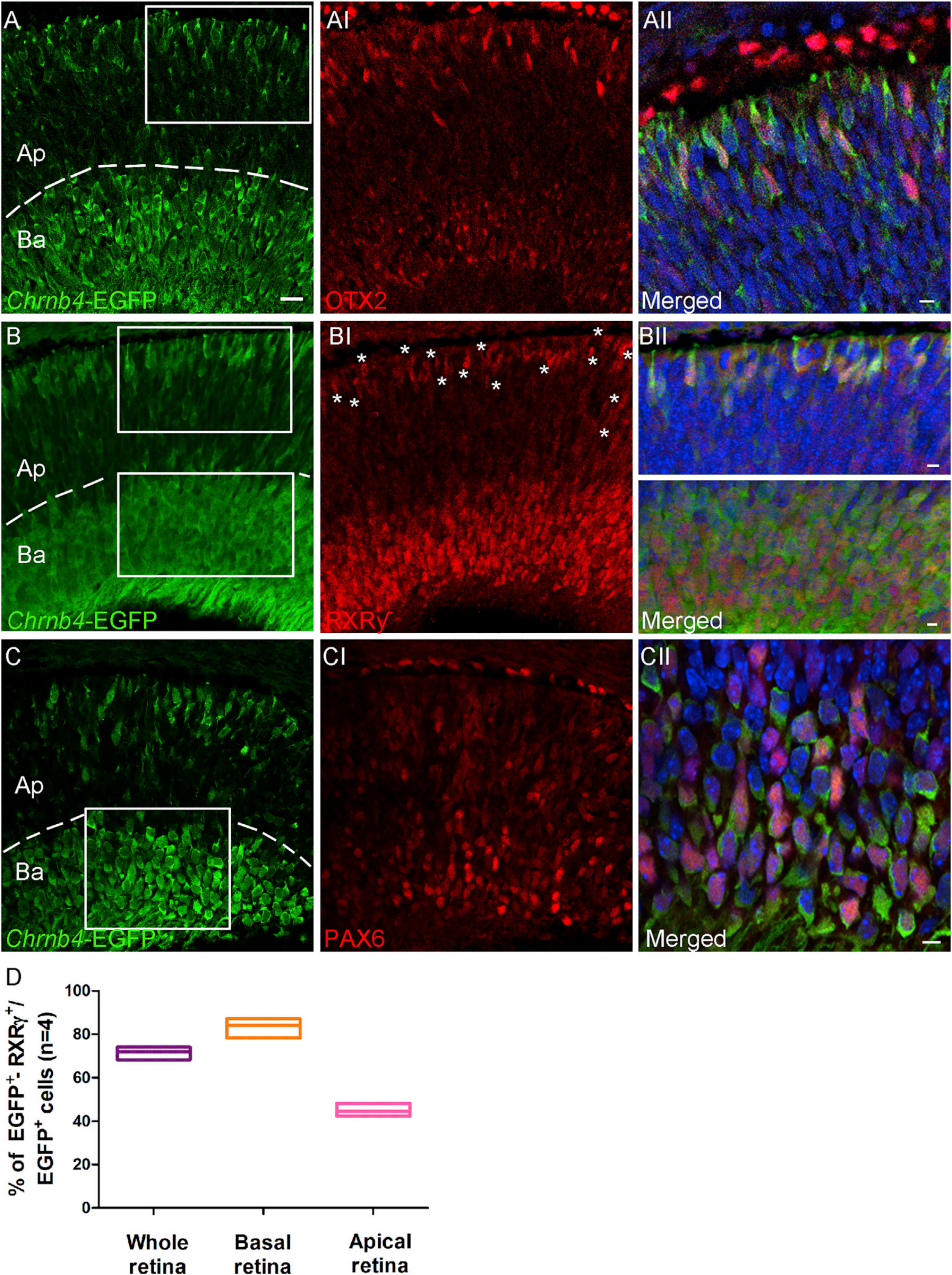
EGFP Co-expression with the Post-mitotic Cone Marker RXRγ at E12 (A–CII) Transversal sections of E12 retinas from *Chrnb4-*EGFP reporter mice. (A–AII) OTX2, essential for photoreceptor differentiation, is detected in elongated EGFP-positive cells adjacent to the apical retinal region. (B–BII) Double RXRγ- EGFP-positive cones localized at both the apical and basal side of the retina. (C–CII) EGFP co-expression with PAX6-positive cells adjacent to the basal retinal region is shown. (A–C) Dashed lines delimit the Ap from the Ba retinal region used for counting in (D). (D) Percentage of double EGFP- RXRγ-positive cells were counted over the EGFP-positive cells detected in the whole, apical-central, and basal retina. Error bars correspond to SEM. (AII, BII, and CII) High-magnification pictures show the corresponding white squares. DAPI, nuclear staining (blue); Ba, basal; Ap, apical; n = 3. Scale bars, 50 μm (A–CI) and 15 μm (magnified insets).

To corroborate the analysis performed on embryonic *Chrnb4*-EGFP retinal sections and to better ascertain the cone identity of a subpopulation of EGFP-positive cells, double-staining immunochemistry and cell transplantations into adult retinas were performed. At E12 the EGFP-positive cells at the outermost (apical) retinal surface co-localize with the transcription factor OTX2 (Figures 3A–3AII) expressed in post-mitotic and photoreceptor precursors at this age. Importantly, almost all the OTX2-positive cells were EGFP positive. Only rare OTX2-positive cells were EGFP negative (0.5% ± 0.2%). At this age, among the EGFP-positive cells, 38.2% ± 4% were OTX2 positive. These results suggest a competence of EGFP-positive cells to give rise to a population of photoreceptor cells.

As the most abundant photoreceptor cell types generated at this developmental stage are cones, a specific cone labeling for the nuclear RXRγ was performed. RXRγ is involved in cone development and is responsible for the S-OPSIN gradient formation. Importantly, RXRγ expression in cones is downstream of OTX2 and OC1 expression.^11^ After stainings, one apical (cones) and one basal (ganglion cells) cell population were identified to be RXRγ positive, as previously described^29^ (Figures 3B–3BII). At the most apical side, where many cones are terminally differentiating, a few bright EGFP-positive spindled cells started expressing the nuclear receptor RXRγ (Figure 3D; 44.6% ± 2.8%). At the basal side, where retinal progenitors and newborn ganglion, horizontal, and amacrine cells are localized, 84.2% ± 3.5% of EGFP-positive cells expressed RXRγ (Figure 3D).

Among the EGFP-positive cells located close to the vitreous (the apical side), 37.1% ± 2% were positive at E12 for PAX6, an early marker of ganglion, amacrine, and retinal progenitors (Figures 3C–3CII; see Figure 2).

In summary, a large population of the RXRγ-positive cells was found to be EGFP positive, indicating that CHRNB4 is present at the beginning of cone genesis (Figure 3) from the early progenitors to cells expressing cone markers.

### Transplanted E12-Derived EGFP-Positive Cells Generate Cones after the Formation of a Secondary Retinal-like Tissue

**Figure 4:**
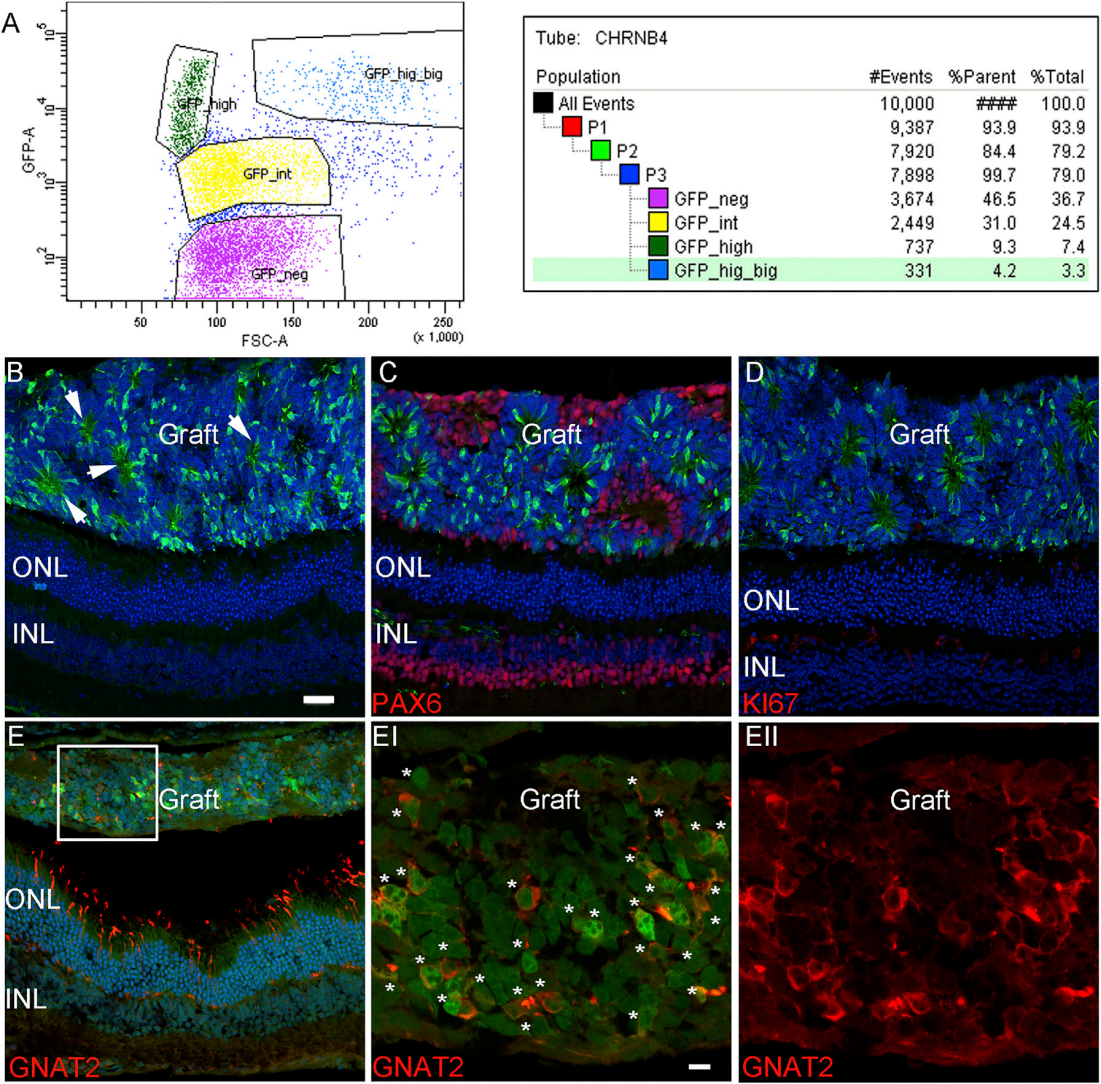
E12-Derived EGFP-Positive Cells Generate Cones after Transplantation into Adult NOD/SCID Retinas (A) Flow cytometry analysis of EGFP-positive cells from dissociated E12 mouse retinas. Different EGFP intensities and cell complexities were detected and gated in blue, green, and yellow, respectively. All EGFP-positive cells were injected. (B–E) Example is shown of rosette-like structures formed in the sub-retinal space of NOD/SCID mice, 4 weeks after grafting (white arrows in B). (C) PAX6-positive (in red) and EGFP-negative cells in the sub-retinal space of adult retinas are shown. (D) Transplanted cells resulted to be KI67 negative. (E–EII) Double GNAT2- EGFP-positive cone cells. (EI–II) High magnification of cell graft is shown (E, white square). DAPI, nuclear staining (blue); ONL and INL, outer and inner nuclear layer; n = 5. Scale bars, 30 μm (B–E) and 15 μm (EI–II).

Taking advantage of cell transplantation as a means to investigate the potential of a specific retinal cell subpopulation to differentiate into adult retinal cell types, 2 × 10^5^ EGFP-positive cells collected from E12 retinas were sorted by fluorescence-activated cell sorting (FACS), and they were transplanted into the sub-retinal space of NOD/SCID adult mice. Immunodeficient adult recipient mice were selected to avoid the potential influence of immune cytokines on cell commitment, survival, and integration. Grafted cells were analyzed 4 weeks after transplantation. A first analysis of the FACS dot plot (Figure 4A) confirmed the previous immunostaining assessment revealing at least three different EGFP-positive cell populations showing a different body size and EGFP intensity. Because of the small retina size and, consequently, the small percentage of cells of interest at E12, all the EGFP-positive cells were sorted via FACS and transplanted. Rare EGFP-positive cells were observed in the recipient retina (data not shown). The most part of transplanted cells aggregated in the sub-retinal space, forming a consistently well-organized cell graft with rosettes (Figures 4B–4EII). The thickness and size of the graft indicated probable cell proliferation, and the EGFP-positive rosette formation suggested the presence of photoreceptors (see white arrows, Figures 4B–4E). The analysis of the cell identity revealed a prevalence of EGFP-positive cell co-labeling of 63% ± 4% with the mature cone-specific marker GNAT2 (Figures 4E–4EII). Another consistent portion of cells (33% ± 6%) was PAX6 positive and EGFP negative, probably mature ganglion or amacrine cells (Figure 4C). Rare cells resulted in being EGFP/KI67 positive, revealing a few progenitors present in the graft at the time of the analysis (Figure 4D).

### The EGFP Expression in the Apical Region of the E15.5 Retinas Is Mainly Localized in Post-mitotic Cone Precursors

**Figure 5:**
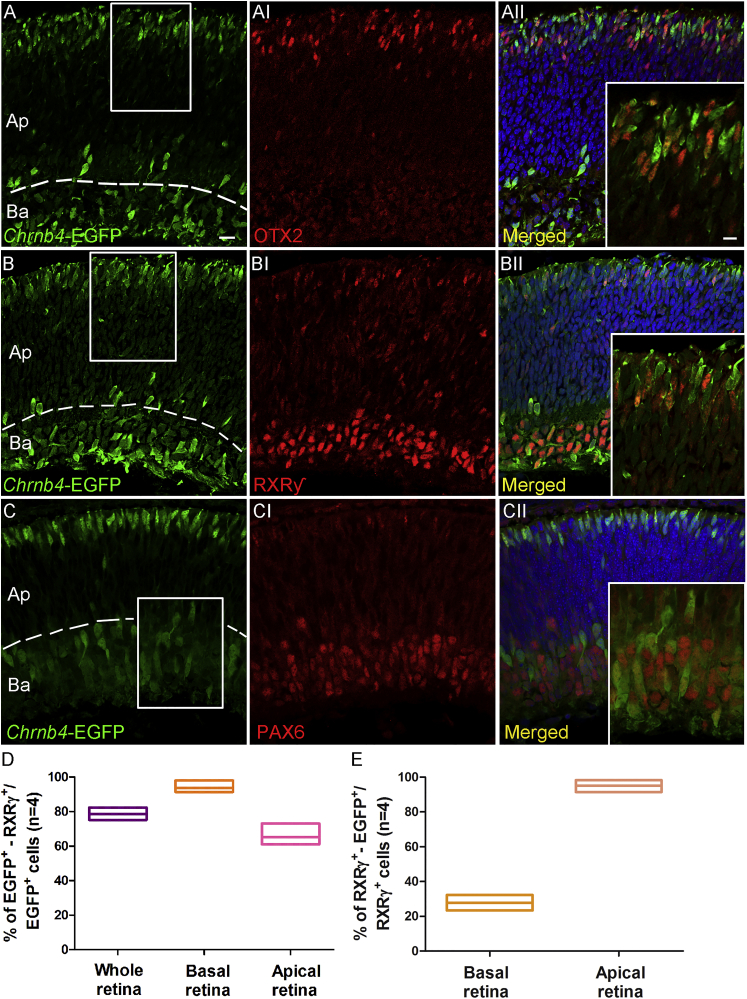
EGFP Expression in Newly Born Post-mitotic RXRγ-Positive Cones of E15.5 Retinas (A–CII) Transversal sections of E15.5 retinas from *Chrnb4-*EGFP mice. (A–AII) OTX2 detection is shown in EGFP-positive photoreceptors at the apical retinal side. (B–BII) Double RXRγ- EGFP-positive cones localized at both the apical and basal sides of the retina. (C–CII) EGFP co-expression with PAX6-positive cells adjacent to the basal retinal side is shown. (D) Percentage of double EGFP- RXRγ-positive cells was counted over the EGFP-positive cells detected in the whole, apical, and basal retina. (E) Percentage of RXRγ^+^-EGFP^+^ was counted over the RXRγ-positive cells detected in the basal and apical-central retina. Error bars correspond to SEM. DAPI, nuclear staining (blue); Ba, basal; Ap, apical; n = 4. Scale bars, 20 μm (A–CII) and 8 μm (magnified pictures in insets).

At E15.5, around one-third of the EGFP-positive cells were confined to the apical region of the retina, presenting an elongated cell morphology typical of cones, whereas the remaining EGFP-positive cells were located in the basal region of the retina (Figure 5). To confirm the cone identity of the EGFP-positive cells, *Chrnb4*-EGFP retinal sections from E15.5 embryos were analyzed for the EGFP co-expression with early cone type-specific markers.

In the apical area, almost 84% (83.8% ± 5%) of the OTX2-positive cells were EGFP positive (low and high intensities). We hypothesized that they are cones in view of the marker expression and cell localization (see Figures 5A–5AII). To confirm the cone identity of the EGFP-positive cells, E15.5 retinas were labeled for RXRγ. At the apical region (Ap) the vast majority of the EGFP-positive cells, around 65%, were positive for RXRγ and only rare RXRγ-positive were EGFP negative, suggesting that the *Chrnb4*-EGFP line traces early-born cones in the E15.5 retinas (Figures 5D and 5E). The small portion of EGFP-positive and RXRγ-negative cells at the apical side might be cone precursors not expressing RXRγ yet. Significantly, from E17 onward, all the EGFP-positive cells were OTX2/RXRγ positive as well as PAX6/Ki67 negative, and transgenic cells adopted a characteristic morphology of nascent cone photoreceptors showing the full elongation of the inner segment (data not shown; see Figures 1D–1F).

At E15.5 at the basal side, 93% of the EGFP-positive cells expressed RXRγ and ∼72% of the RXRγ-positive cells were EGFP negative (Figures 5B–5BII and 5D). A few PAX6/EGFP-positive cells were still detectable at the basal side of the retina, which could be either precursors of amacrine or retinal ganglion cells.

These results prove the competence of the *Chrnb4*-EGFP line to trace cone generation from progenitors to post-mitotic cells. Furthermore, this line enables transient tracing, up to E15, of newborn PAX6-positive cells in the retinal basal region, where ganglion, horizontal, and amacrine cells are generated.

### Presumptive Cone Precursors Collected from E15.5 *Chrnb4*-EGFP Retinas and Transplanted into the Adult Retina Exchange Cone-Specific Materials between the Donor and the Host Cells

**Figure 6:**
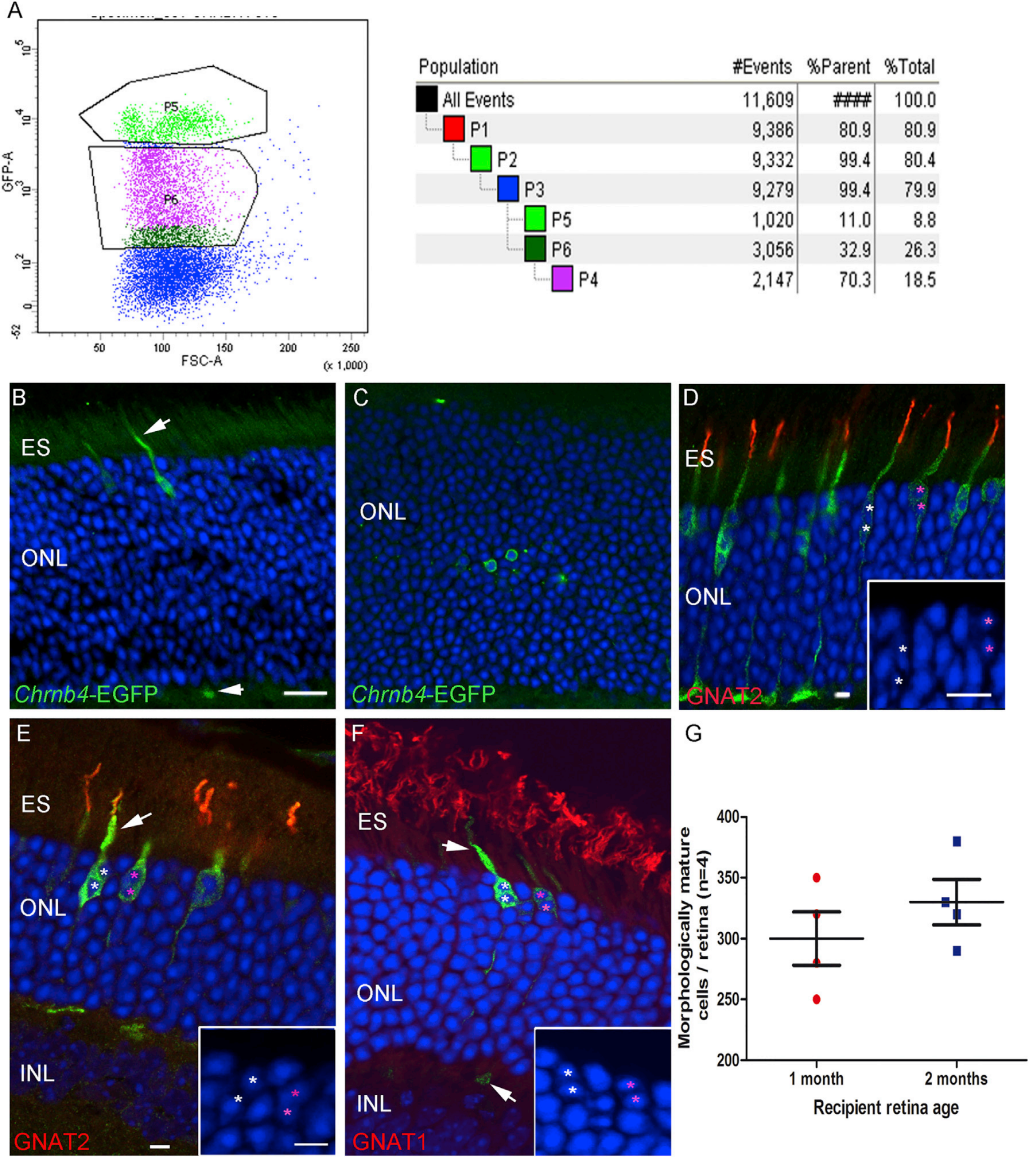
Presumptive Cone Precursors and Newborn Cones from E15.5 *Chrnb4*-EGFP Retinas Generate the Appearance of EGFP-Positive Mature Cones after Transplantation into Adult NOD/SCID Retinas (A) Flow cytometry analysis of *Chrnb4*-EGFP-positive cell populations from dissociated E15.5 mouse retinas. Only the cells presenting the highest EGFP expression (P5 gated cells, in green) were transplanted. (B and C) Examples show (B) mature and (C) immature cone photoreceptors detected in adult recipient retinas. Note the presence of outer segments (white arrow, B). (D) Example is shown of a 2-month-old *Chrnb4*-EGFP-positive mouse retina stained for GNAT2 as control staining. Mature GNAT2-positive (E) and GNAT1-negative cones (F) 4 weeks after transplantation into NOD/SCID mice are shown. (B, E, and F) White arrows indicate external segment or synapsis. (G) Quantification is shown of morphologically mature cells observed in the adult retinas defined by the presence of the external segment or synapsis-like morphology. DAPI, nuclear staining (blue); ONL and INL, outer and inner nuclear layer; ES, external segment; n = 4 per experiment. (D) No significant variations of cell integration were revealed with two different ages of mouse recipients, n = 3. Error bars correspond to SEM. Scale bars, 20 μm (B and C) and 5 μm (D–F insets).

As the previous results highlight that the *Chrnb4*-EGFP is present in post-mitotic newborn RXRγ-positive cones in E15.5 retinas, the potential of the brightest EGFP-positive cells to give rise to adult mature cones was investigated by transplantation. The retina at E15.5 contains more cells than at E12.5, allowing the selection of different EGFP-intensity cell subpopulations. The examination of the FACS dot plot (Figure 6A; Figures S3A–S3AI) confirmed the previous immunostaining assessment revealing two main cell populations showing a different EGFP intensity, and this led us to hypothesize that the most intense EGFP-positive cells would contain cone precursors. This was first validated by gene expression qPCR analyses revealing the presence of mRNAs for *Crx*, *Onecut1*, *Rxrγ*, and *Trb2*. The cell selection also included cells expressing the *Brn3b* genes specific for retinal ganglion cells (RGCs) and *Calbindin* present in the horizontal cells (Figure S3B). The 2 × 10^5^ EGFP-positive bright cells, as those gated in Figure 6A (FACS dot plot; P5, light green cells), were thus collected and transplanted into the sub-retinal space of adult NOD/SCID mice. This population represented around 9% of the total retinal cells, and it seemed to contain two cell populations of different size and granularity (Figure 6A).

Then 4 weeks after transplantation into NOD/SCID retinas, the results of E15.5-donor cell transplantation revealed a marked difference in the expression pattern of the EGFP in the adult recipient retinas compared to E12 cells. As a first evaluation, the success of cell transplantation into adult recipient retinas was decreed after the detection of the external segment or of a seemingly synaptic process (Figures 6B, 6E, and 6F, white arrows). Cells with no processes were not included in the counting (Figure 6C). Similar results were obtained in the 1- and 2-month-old NOD/SCID mouse retinas (Figure 6D). Some EGFP-positive cells showed a complete external segment, a synapsis morphology, and a chromatin condensation characteristic of cone cells (Figures 6B, 6E, and 6F).

Of the EGFP-positive cells detected in the ONL of the recipient retinas, 89% were positive for GNAT2 (cones) and 100% negative for GNAT1 (rods), underlining the propensity of the bright EGFP-positive cells collected from E15.5 retinas to give rise to cells expressing mature cone markers only (Figures 6E and 6F). The most part of the grafted cells stacked in the sub-retinal space expressed markers of adult cones, such as GNAT2, without showing a fully mature morphology (data not shown). A minor part of them (3.4% ± 2%) were positive for PAX6 and all were negative for the proliferation marker Ki67 (data not shown).

As rod transplantation into certain models of retinal dystrophies with diminished functional rods resulted in an improved migration and integration of rods,^30^ we hypothesized that an environment in which cones are not functional might further favor the cone migration and integration into recipient retinas. To enhance cell integration into the host retina, E15.5 bright EGFP-positive immature cones were injected into the sub-retinal space of a retinal model of cone-rod dystrophy, namely the *Cnga3*^−/−^ mouse line.^31^ Since different features occurring during the disease progression have been shown to diminish the transplanted cell migration efficiency^30^, ^32^ by developing physical barriers, such as high glia scarring and maintaining a steady outer limiting membrane (OLM) integrity, recipient mice were screened for the presence of these barriers before transplantation (Figure S4). In view of the low gliosis and the OLM disruption observed, the best recipient stage was identified to be 2-month-old *Cnga3*^−/−^ mice (Figure S4). Indeed, after the grafting of E15.5 highly EGFP-positive cells into the 2-month-old recipient retinas, several EGFP-positive cells were found with a mature cone morphology and GNAT2 protein expression (data not shown). Their number was similar to the result achieved with the NOD/SCID recipient line. A very limited amount of EGFP-positive cells were present in the retina ONLs of 1- and 3-month-old mice (Figure S6).

As the mouse retina consists mainly of rods, 97% versus 3% of cones in the ONL, we hypothesized that transplantation into a cone-enriched mouse retina, the *Nrl*^−/−^ mouse line,^33^ might provide a more appropriate cone-like environment, favoring the cone precursor migration and differentiation. The *Nrl*^−/−^;*Rpe65*^*R91W*/*R91W*^ mouse line^34^ with a less dysmorphic retina was preferred to the rosette-forming *Nrl*^−/−^ line.^35^ Age-related progression of gliosis was observed in all the retinal stages examined (Figures S5B–S5BII, S5D–S5DII, and S5F–S5FII), as well as a disruption of the OLM integrity (Figures S5A–S5AII, S5C–S5CII, and S5E–S5EII). Only a few EGFP-positive cells with proper photoreceptor morphology were detected in retinas of this mouse line as compared to the previous transplantations (Figure 6; Figure S6).

Then, because EGFP expression is much more restricted to the presumptive cone population at E17.5 and P1 in the transgenic line, the capacity of these EGFP-positive cells to generate cones in vivo after transplantation also was explored. However, only very few cells were detected in the retinas after transplantation (data not shown).

**Figure 7:**
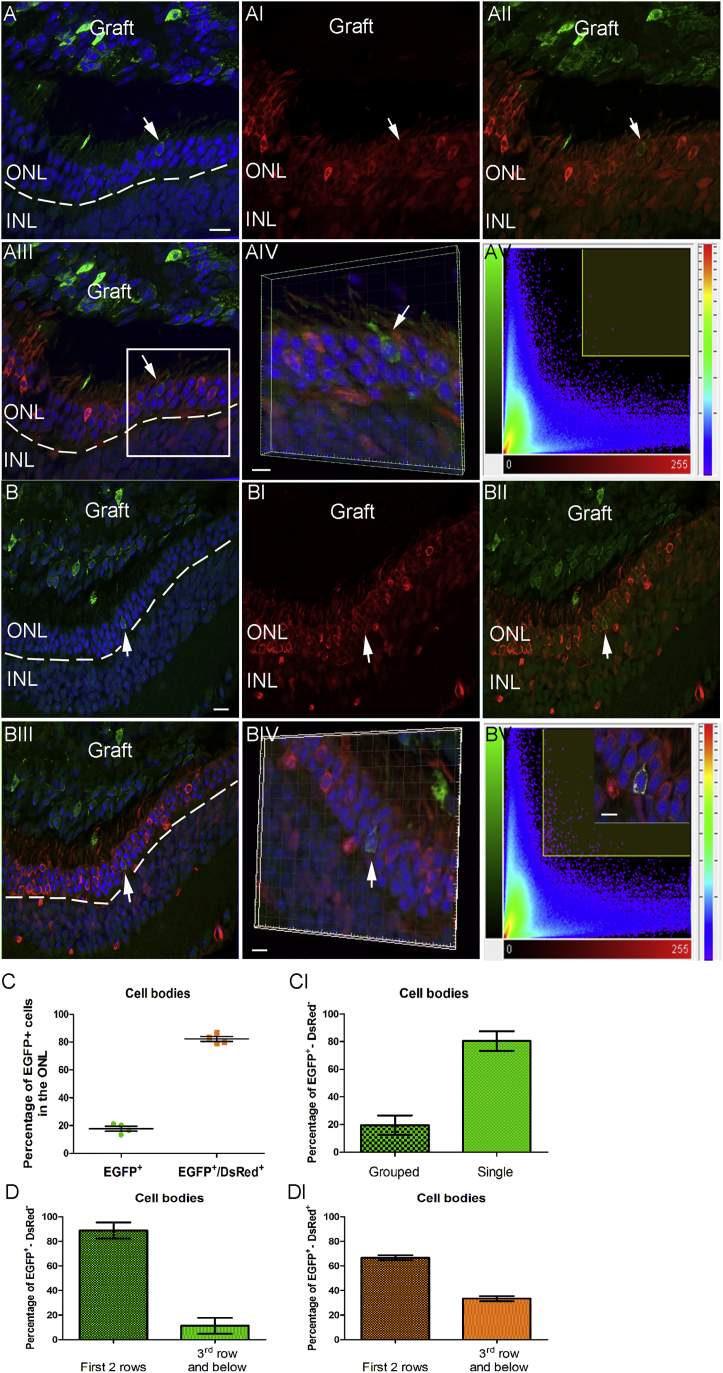
Evidences of Donor and Host Cell Material Exchanges after the Transplantation of E15.5 Chrnb4-EGFP Cells into the Adult DsRed Retinas (A–BV) Transversal sections of adult DsRed retinas transplanted with E15.5 *Chrnb4-*EGFP positive cells. (A–AV) An example is shown of a transplanted cell present in the host retina expressing only EGFP analyzed by the Manders’overlap coefficient visualized in the image as white spots. (B–BV) The same analyses were performed in a transplanted cell expressing EGFP and the host marker DsRed pointed out by the white arrow (BIV–V). (C–DI) Quantification of single- and double-labeled cells reveals that the large majority of EGFP-positive cells also express the host DsRed transgene reporter marker (C) and are dispersed in the ONL with a preference for the first two rows of photoreceptors (DI). EGFP-positive cells not containing DsRed are usually isolated (CI) and are also mainly located in the two first rows of photoreceptors of the outer part of the ONL (D). Error bars correspond to SEM. DAPI, nuclear staining (blue); ONL and INL, outer and inner nuclear layer; n = 6 injected mice. Scale bars, 15 μm (A–AII and B–BIII) and 5 μm (AIV and BIV–BV).

During our analysis of transplanted cells, we noticed that some EGFP-positive cells had an elongated nucleus (see Figure 6), which also was observed in the adult retinas of *Chrnb4*-EGFP mice (see Figures S2C–S2CII). Nonetheless, to investigate the possibility of cell fusion, we transplanted the E15.5 EGFP-positive cells into a DsRed transgenic mouse line retina, which ubiquitously expresses the red fluorescent protein^36^ (Y.A., unpublished data). Surprisingly, the vast majority (>80%) of the EGFP-positive cells present in the retina also were positive for the host DsRed transgene (Figure 7). Interestingly, almost all the double-positive cells were grouped, whereas the cells expressing only EGFP were in general detected as single cells (Figure 7CI). The single EGFP-positive cells were located with a large preference (around 80%, Figure 7D) in the two rows at the apical side of the ONL (close to the graft). The double-positive cells were more diffusely distributed into the ONL but also with a preferred location in the two first rows in the vicinity of the graft.

Surprisingly, double-positive cells also were present inside the graft, showing that the exchanges of cell materials occurred also from the host toward the graft, highlighting the occurrence of a bidirectional material transfer between donor and recipient cells.

### Chrnb4-EGFP Expression Is Restricted to All Cone Types in the Adult Mouse Retina

**Figure 8:**
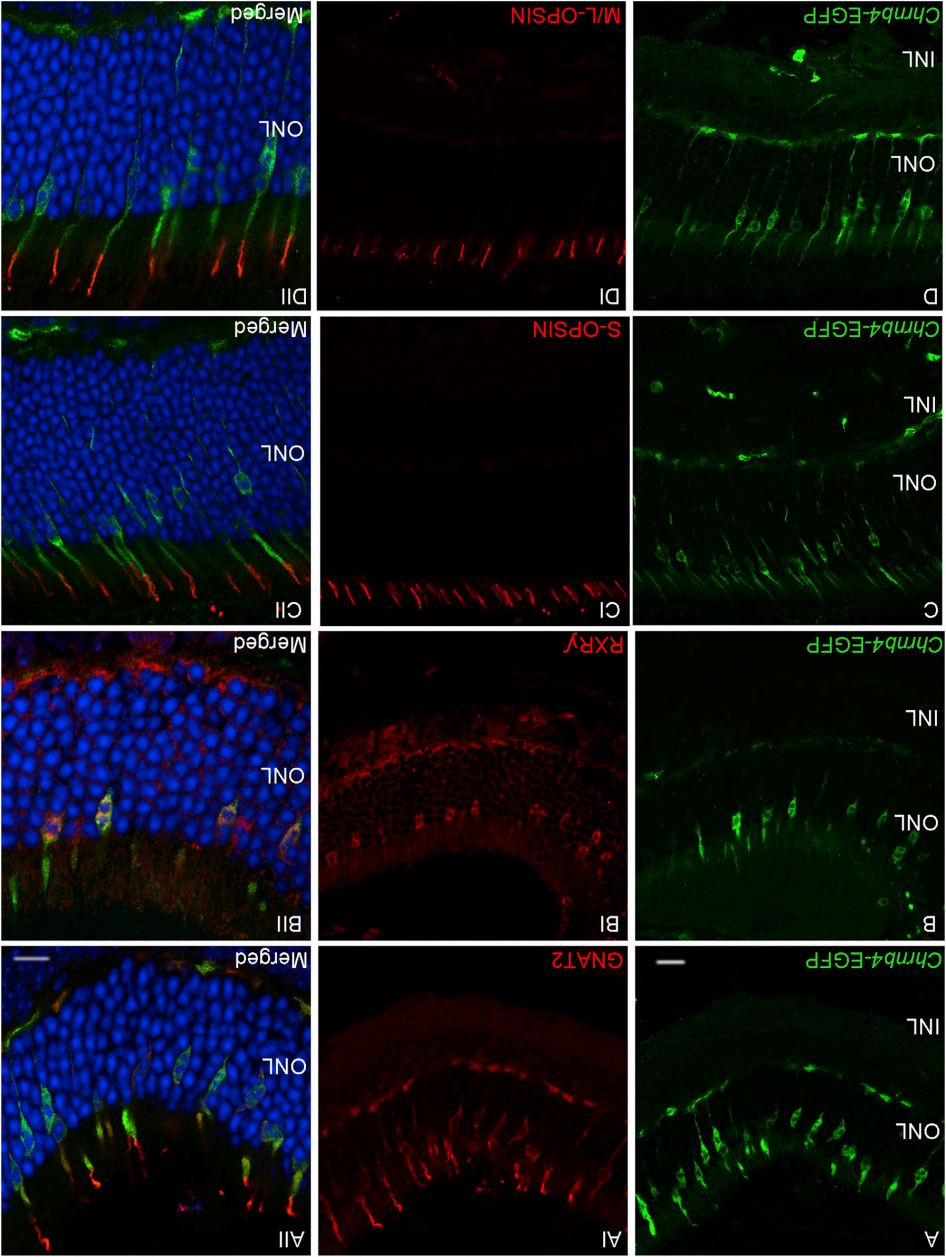
*Chrnb4*-EGFP Is Expressed in All Cone Subtypes of Adult Mouse Retinas (A–DII) Transversal sections of *Chrnb4*-EGFP mouse adult retinas. EGFP co-localization is shown with cone-specific proteins, such as GNAT2 (AII), RXRγ (BII), S-OPSIN (CII), and M/L-OPSIN (DII) (red labelings). The column AII–DII is a magnification of pictures from A to DI. ONL and INL, outer and inner nuclear layer; DAPI, nuclear staining (blue). Scale bars, 10 μm (A–D and AI–DI) and 20 μm (AII–CII).

As previously described by Siegert and colleagues, the Chrnb4-EGFP reporter gene expression was first identified in adult mouse retinas for its co-localization with the peanut agglutinin (PNA) antibody,^20^ a marker of cone photoreceptors. Since the two different cone subtypes, the medium/long wavelength- and the short wavelength-sensitive cones, are generated before birth but become mature only at postnatal stages, the EGFP expression in different cone subtypes was examined by M/L- and S-OPSIN stainings in adult retinas. Notably, in adult retinas the EGFP expression intensity decreased over time, as confirmed by the FACS analysis and by the retinal sections not stained with the EGFP antibody (both not shown). The EGFP-positive cells expressed the pan-cone markers GNAT2 and RXRγ (Figures 8A–8BII) as well as the short and the long wavelength proteins S-OPSIN and M/L-OPSIN, respectively (Figures 8C–8DII), highlighting the fact that the *Chrnb4*-EGFP line traces both cone subtypes.

### Detection of the CHRNB4 Protein in Human Cones

To verify whether the expression of CHRNB4 also may serve to follow human cone development, the endogenous expression of the CHRNB4 receptor subunit was studied in fetal and adult human retinas by immunofluorescence and 3,3’-diaminobenzidine (DAB) labeling as well as by RT-PCR on adult retina extracts. In 3-month-old fetal retinas, similarly to the developing mouse counterpart at E13–E14, the native CHRNB4 protein was revealed at the cellular periphery of most retinal cells, with a higher expression in cells adjacent to the basal and to the apical retinal surfaces (Figures S8A–S8AI). At the retinal basal side, some putative ganglion cells and elongated progenitors were CHRNB4 positive (Figures S8A and S8AII). The control retinas used in these experiments, incubated without the primary CHRNB4 antibody, did not show any aspecific signal (Figure S8B).

Different adult human post-mortem retinas from donors aged 69–84 were analyzed by RT-PCR for their content of *CHRNB4* mRNA. As shown in Figure 8C, all the examined samples were *CHRNB4* positive. CHRNB4 protein was mostly detected in pyramid-shaped inner segments of cone external segments, and it co-localized with the pan-cone-specific GNAT2 proteins (Figures S8D–S8DII).

These results considered all together define *CHRNB4* as a suitable tracer of mouse and human cone development, opening the avenue to future studies aiming to shed light on mechanisms regulating cone development and degeneration. The expression of CHRNB4 also can serve to optimize cone transplantation in the perspective of retina repair.

## Discussion

In this study, we provide a characterization of the *Chrnb4*-EGFP mouse line in the retina, revealing the progressive specific localization modifications of the reporter gene during development. The EGFP is detected in early progenitors, then probably in cone precursors as well as in other early-generated retinal neuron precursors, up to E15.5 (not yet demonstrated). After, the EGFP expression becomes progressively restricted to adult mature cones. This pattern of expression, which can be easily traced with this transgenic line, opens new perspectives for developmental studies of the retina and cone specification.

The transplantation studies in both healthy and diseased retinas confirm the fate determination of the *Chrnb4*-EGFP-positive cells isolated at different developmental stages. These experiments allow the identification of a cone precursor population more competent for cell/material transfer than those described so far, and they reveal the importance of identifying this population well in order to optimize the grafting procedure. Our studies also reveal that the great majority of cones interpreted until now to have integrated the recipient retina after transplantation are, in fact, the results of cell material exchanges/fusion. These different topics are discussed below.

Cell fate acquisition in the retina is a complex integration of different signals determined autonomously as well as with input from the environment. Transcription factors^13^, ^27^ and genes involved in cell proliferation regulation interact at different stages to control the number of cells in a given cell population and to generate specific cell phenotypes.^37^, ^38^, ^39^, ^40^ Although some genes are common to different species, some variations were noted among fishes, frogs, birds, and mammalians.^27^ Lineage studies demonstrated that, at least in mouse, progenitors are not limited to producing one single type of retinal neuron. Indeed, the isolation or the tracing of retinal progenitors from different stages of development further demonstrated that the probability of generating specific fates in vitro and in vivo changes over the period of neurogenesis.^28^, ^41^, ^42^ Recent studies also demonstrated that the non-differentiated retinal cell pool is composed of distinctive types of multipotent progenitors and precursors, which show biases in the production of subsets of retinal neurons during the terminal division. Such precursors undergoing terminal division are Olig2 positive.^8^

In this context, transgenic mouse lines for which specific cell types can be traced during development are important tools to study different aspects of the retinogenesis and to better characterize the different cell stages throughout the development.

Several important studies have allowed the identification as well as the characterization of different cell precursors during retinogenesis,^13^, ^27^ and various molecular pathways also were described concerning certain cell fate acquisitions. Some molecules were documented as key actors for cone fate acquisition, such as OTX2 and OC1^11^ and 2, whereas other transcription factors served as markers to identify precursors involved in cone generation, such as Olig2 (which seems to provoke cell-cycle arrest^8^). Until now, the molecular basis of cone fate commitment from the progenitor to the cone precursor was characterized by the expression of OLIG2, OTX2, and OC1, which still remains unclear because of the difficulty to select subpopulations of retinal progenitor cells undergoing this transition.

The present mouse line may contribute to such studies by raising the opportunity to isolate early progenitors becoming progressively restricted to the cone fate. This transgenic mouse offers a sort of red line to follow during cone development. Indeed at E12–E13, not all proliferative cells revealed by EdU labeling are positive for EGFP, indicating that the *Chrnb4*-EGFP line highlights only a subpopulation of retinal progenitor cells in the early developmental stage. Moreover, the late progenitors are not EGFP positive. In addition, the FACS analysis we performed revealed different EGFP-positive cell populations at E12–E13. Cones are the most abundant cell type generated between E14 and E15.^26^ Since after E15 rare proliferating cells are EGFP positive, we may predict that at early developmental stages a population of EGFP-positive progenitors is fated to become cones. Indeed, these cells migrate toward the apical region, become post-mitotic, and then express the cone marker RXRγ. These various EGFP patterns of expression and diverse populations of progenitors expressing CHRNB4 give a new opportunity to study the progressive fate restriction in retinal progenitors giving rise to cones for instance. Therefore, this line might be a tool to acquire a better knowledge of the molecular mechanisms involved in the early retinal progenitor to cone precursor program shift. Interestingly, the pattern of CHRNB4 expression is similar in the human retina, suggesting that the tracing of CHRNB4 also may help to study the mechanisms leading to human cone formation.

A similar approach can be envisaged to study amacrine cell, horizontal cell, and RGC formation; although our study was not focused on these cell types, the pattern of proliferating and post-mitotic EGFP expression in the basal region is compatible with the generation of such cells. At E12.5, EGFP-positive cells localized in the middle and basal retina incorporated EdU and expressed the PAX6 protein. These results suggest the presence of progenitors and newborn ganglion, horizontal, or amacrine cells in a subpopulation of EGFP-positive cells in this region. Deeper analyses are needed to better characterize the different early cell subtypes traced by the *Chrnb4-*EGFP line.

Cell transplantation was used to better characterize different subpopulations of cells expressing CHRNB4 at various developmental stages. The injection of E12 EGFP-positive cells gave rise, in the sub-retinal space, to a large retina tissue organized in rosettes containing cells expressing the cone marker GNAT2, and, in certain rosettes, the transcription factor PAX6. These experiments confirm that this EGFP-positive population contains retinal progenitors and/or precursors able to generate at least cones.

Concerning retina repair by cell transplantation, the E15.5, but not the E12, retina-derived EGFP-positive cells grafted in NOD/SCID adult retinas gave rise to EGFP-positive cells expressing the adult cone markers GNAT2 in the ONL of the recipient retinas. Interestingly, in a previous work aiming to identify the most competent cell population to form rods after transplantation, MacLaren et al. observed that retinal progenitors have a very low capacity to integrate the retina, whereas newly post-mitotic rod precursor expressing GFP, grafted into a rod function-deficient mouse, resulted in GFP-positive photoreceptors with functional synapses.^43^ Our data are in accordance with these results, and the E12-derived EGFP-positive cells appeared even more refractive to interact with the retina.

The cells expressing EGFP intensely, collected at E15.5, showed the competence of a specific population to transfer cone-specific signatures into the ONL of adult recipient retinas after transplantation. This confirms that the *Chrnb4*-EGFP-expressing cells at this stage are indeed either committed to forming cones or are newly born cones. Interestingly, Lakowski et al.^44^ also observed that donor cells selected for their expression of GFP under the control of the *Crx* promoter, activated both in cones and rods, contain more GFP cells in the recipient retina when isolated between E14.5 and E17.5 than those selected at an earlier or older age. However, in our study, the number of EGFP-positive cells detected in the recipient retina was ten to 15 times higher than in the *Crx*-GFP transplantation studies. Our results suggest that, with the parameters applied to the cell sorting, we caught another cell population less mature than the CRX-expressing cells or mixed populations containing immature cells and some CRX-positive cells. Our data also suggest that the integration competence (cell or material, see below) may occur while the cone precursor is still migrating or just finishing its course upon reaching the apical region and probably also during the last division, when the fate decision is just launched for cone formation.^8^, ^11^ Such characteristics thus seem to be essential to exchange the adequate material between the grafted cells and the host retina after transplantation. Some non-mature EGFP-positive cells were observed in the center of the retina and thus support the idea of having isolated immature precursor cells, which had not yet launched appropriately the cone differentiation program.

Nonetheless, the high number of host cells simultaneously expressing DsRed and the EGFP protein demonstrates material exchanges between donor cells and the recipient retina, raising important concerns about cell transplantation effectiveness. The analysis of all EGFP-positive cells in the retina reveals that 89% of them express the cone marker GNAT2, whereas the rod marker GNAT1 is absent. It is also important to consider that host material or cells also were found in the sub-retinal graft, disclosing bidirectional exchanges of cellular materials (transplant toward the retina and the retina toward the transplant). In addition, many EGFP cells were located in the normal cone localization (the apical part of the ONL). This suggests at least two main possible explanations.

In the first hypothesis, the donor cells fuse part of their cytoplasm or release diverse materials that are internalized by the host cells, and they completely change the cell program of rods to convert them into cones. This at the first glance seems highly unlikely, knowing the successive events necessary for cone commitment^13^, ^27^ and the epigenetic modifications occurring in rods and cones after their differentiation.^45^, ^46^ It was, nonetheless, recently shown that the downregulation of NRL in the adult retina provokes a cone-like phenotype with rod characteristic defects.^47^ It is puzzling that we did not find cells expressing both rod and cone markers or rod markers in the EGFP cells if material is exchanged between these two types of cells. If reprogramming is truly triggered by the donor cells, this opens a very important field of investigation. The second possible explanation is that cone precursors exchanged materials only with endogenous cones. The observation of many EGFP-positive cells in locations where cones normally reside supports this hypothesis and appears the most probable. This suggests that the cellular material transfer, like exosomes, for instance, occurs through specific proteins or molecules, allowing the recognition of cones. In both hypotheses, the cell transplantation remains valid to pursue in order to enhance retina repair by either material exchanges or by true cell integration. This is a new field of investigation that will necessitate the understanding of the different mechanisms controlling the material transfer between donor and host cells.

The fact that only a particular developmental stage of the maturing cone has the capability to transfer material or to integrate in the retina should help to reveal which cell function is responsible for this exchange and which material is crucial for this phenomenon. The cone appears to be an ideal cell type to study transplantation into the retina, because it allows one to better evaluate which material is exchanged and in which direction. Rod transplantation into a rod-dominant retina would make it difficult for one to study the material and molecule exchanges between cells and to test the reprogramming hypothesis.

During the revision of this work, two important papers were published side by side revealing the material exchanges between host and donor cells using rod precursors (Nrl-EGFP mouse) or newly generated photoreceptors (*Crx*-GFP embryonic cell line). Sub-retinal transplantations into DsRed mice were performed. In both studies, around 80% of the EGFP-positive cells were also DsRed positive, as evidenced by FACS and immunohistochemistry investigations.^48^, ^49^ Such observations were confirmed using Cre-expressing cells injected into the tdTomato retina, which has a stop codon after the CAG ubiquitous promoter.^48^, ^49^ Interestingly, fluorescence in situ hybridization (FISH) studies of male donor cells into female hosts revealed no cell nucleus fusion but cellular material exchanges.^48^, ^49^ Injection of recombinant proteins did not explain these exchanges.^48^ These studies demonstrate that a specific cell stage of the rod photoreceptor development allows material exchanges between donor and host cells, creating a new perspective to transfer therapeutic materials into a diseased organism. Indeed, the transplantation of healthy rods into a *Gnat1*^−/−^ deficient retina results in the expression of the Rod-α-transducin protein in the host retina.^48^ In these studies, no indications on cone behavior characteristics were documented, and, thus, the *Chrnb4*-EGFP mouse line should help to understand the mechanisms involved in the cell exchanges. It is, nonetheless, remarkable to note that in our study and the two mentioned above, a similar number of double-positive cells (80%) was observed, although the cell populations studied were different. In view of the FISH data in both cited studies, it is highly probable that almost all the transplanted cones did not integrate into the retina but transferred crucial materials to the host (cone) cells. Future experiments necessitate deep characterization and compilation of studies in rods and cones.

As previously observed for rod precursor integration, the transplantation efficacy varied markedly depending on the stage of the disease.^30^, ^50^, ^51^ It was shown that gliosis may impair the grafted cell interaction with the retina,^32^ but not in every case,^30^ whereas a disruption of the OLM integrity facilitates donor cell exchanges with the host retina.^50^ In our study using the *Nrl*^−/−^;*Rpe65*^*R91W*/*R91W*^ line, composed of cone-like cells, although poorly efficient in this model, the best-transplanted cell material interactions occurred when gliosis was low and the OLM rupture was high. A similar observation was made with the *Cnga3*^−/−^ cone dystrophic mouse model that we used. But, unlike a previous publication^44^ describing a marked transplantation improvement using E15.5-derived *Crx*-GFP cells injected into the *Gucy2e*^−/−^ cone dystrophic mouse retina, no augmentation of the number of EGFP-positive cells was observed in the retina of *Cnga3*^−/−^ animals in comparison to NOD/SCID mice. In that case, it is possible that the balance between gliosis and OLM disruption never reached a very favorable situation for cytoplasmic material exchanges.

All these data, with the previous studies cited above, suggest that a particular cone precursor cell stage and favorable environment of the host cells are necessary for the successful treatment.

CHRNB4 is the β4 subunit of the cholinergic receptor and was not described before in the retina; thus, its function remains unknown in this tissue. Nonetheless, this subunit previously was shown to conduct fast synaptic transmissions.^52^ Because cones operate under bright-light conditions, a role of CHRNB4 in the fast cone adaptation to light stimuli may be envisaged. Equally important, its role in retinal progenitors and cell precursors is also a key issue to understand whether neurotransmitter release also may participate in some features of retinogenesis steps, as it was previously described in other systems from stem and progenitor regulation^53^, ^54^ to neuron differentiation.^55^

In conclusion, the *Chrnb4*-EGFP mouse line allows the tracing of a subpopulation of early retinal progenitors that generate cones in the apical retina region and probably retinal ganglion, horizontal, and amacrine cells in the basal part. The progressive restriction of the EGFP expression in cone cells throughout the retinogenesis opens new perspectives to analyze the molecular fate decision occurring during the retinal progenitor to cone precursor program switch. Our transplantation studies reveal that early cone precursors are the most prone to interact with the host retina after transplantation. The characterization of this cell population is crucial to ameliorate the material exchanges in order to preserve the cone cell identity, with the aim of restoring cone function to potentially repair the retina for daylight vision. In that context, the feasibility of daylight vision restoration in a mouse model of cone dystrophy (the *Cpfl1*^−/−^ mouse) was obtained recently, using cone-like cells isolated from the *Nrl*^−/−^ mouse by magnetic-associated cell sorting (MACS) based on the CD73 antigen expression.^56^ Even if cell material exchanges also may have occurred in this study, the proof of principle of cell transplantation to ameliorate day vision was well provided.

## Materials and Methods

### Animals

The NOD/SCID mice used in this work were obtained from Charles River Laboratory, the *Chrnb4*-EGFP mouse line was obtained from the GENSAT project, the *Cnga3*^−/−^ mice were obtained from Martin Biel, and the *Nrl*^−/−^;*Rpe65*^*R91W*/*R91W*^ mice were obtained from Marijana Samardzija (described in Samardzija et al.^34^) The animals were treated according to institutional and national as well as the Association for Research in Vision and in Ophthalmology (ARVO) guidelines. All the experiments as well as the procedures were approved by cantonal veterinary authorities. All mice were kept on the standard 12-hr dark-light cycle.

### FACS Analysis

Retinas were dissociated according to the manufacturer’s instructions using a Papain kit (Worthington Biochemical) at different time points of the retina maturation, and single cells were sorted via FACS for the EGFP expression. Cell sorting was performed using a MoFlo Astrios (Beckman Coulter’s company at the UNIL platform, CHUV), fitted with a 488-nm green laser to excite EGFP.

### Transplantation of In Vitro Retina-Derived Photoreceptors

Adult recipient NOD/SCID, *Cnga3*^−/−^, and *Nrl*^−/−^;*Rpe65*^*R91W*/*R91W*^ mice were anesthetized with a reversible anesthetic regimen composed of Ketamine/Dormitor (Ketamine 30–60 mg/kg, Parker Davis; Dormitor 0.5–1 mg/kg, Graeub) and reversed with the injection of Antisedan (0.5–1 mg/kg, Graeub). Recipient mice were transplanted between 6 and 16 weeks of age. Prior to transplantation, Chrnb4-EGFP-derived retinas at different stages of maturation, from E12 to P1, were dissociated using a papain kit (as suggested in the protocol; Worthington Biochemical) and sorted via FACS for the GFP channel. The 2 × 10^5^ sorted photoreceptors were resuspended in 1 μl sterile Hank’s balanced salt solution (HBSS) with the addition of DNase (0.005%, Worthington Biochemical), and they were injected into the sub-retinal space of adult mice through a Hamilton syringe with a 34G needle (BGB Analytik). Then 4 weeks post-injection, grafted mice were culled down by CO_2_ and the retina analyzed as below.

### Tissue/Cell Fixation and Immunohistochemistry/Immunocytochemistry

Transplanted and non-transplanted retinas at different developmental stages were fixed with 4% paraformaldehyde (PFA) in PBS for 30–60 min at room temperature (RT), bathed in 30% sucrose at least overnight at 4°C, embedded in yazulla for 30 min, and frozen at −20°C before sectioning. The 12-μm sections prepared on Superfrost plus glass slides (Thermo Scientific) were incubated for 1 hr in blocking buffer (0.1%–0.3% Triton X-100; 1%–10% goat, rabbit, or sheep serum; and 0.1–0.5 mg/mL bovine serum albumin [BSA, Sigma-Fluka] diluted in PBS), and they were incubated overnight at 4°C or RT with primary antibodies.

Sections of adult or developing eyes were used for immunohistochemical analysis to confirm antibody specificity. The following antibodies were used: PAX6 (Covance, rabbit, 1:300), OTX2 (Abcam, rabbit, 1:300), S-Opsin (Merck Millipore, rabbit, 1:2,000), ML-Opsin (Merck Millipore, rabbit, 1:2,000), RXRγ (Santa Cruz Biotechnology, rabbit, 1:200), GNAT2 (Santa Cruz Biotechnology, rabbit, 1:200), GNAT1 (Santa Cruz Biotechnology, rabbit, 1:1,000), Zo1 (Invitrogen-Thermo Fisher Scientific, rabbit, 1:2,000), GFAP (Dako Schweiz, rabbit, 1:500), Ki67 (Becton Dickinson, mouse, 1:75), CHRNB4 (Covalab, goat, 1:100), and CHRNB4 (Santa Cruz Biotechnology, goat, 1:100).

After rinsing three times 10 min with PBS, sections were incubated for 1 hr at RT with the appropriate cy2, cy3 Alexa-tagged secondary antibodies (Invitrogen-Thermo Fisher Scientific) and rinsed. Concerning the CHRNB4 antibody staining, an antigen retrieval treatment was performed based on Citric acid (pH 6) prior to the addition of the primary antibodies. For human samples, the horseradish peroxidase (HRP, Vector Laboratories) and the peroxide/DAB substrate (Dako) are recommended, since various fluorochromes tested give rise to a high background. The nuclei were counterstained with DAPI (Sigma-Fluka). Control sections were incubated with secondary antibodies only. Stainings were observed using either a Zeiss (LSM 510) or a confocal (Leika SP5) fluorescence microscope. Click-it EDU kit (Invitrogen-Thermo Fisher Scientific, C10339) was used as recommended by the supplier.

### Quantification

Integrated cells were counted 4 weeks after transplantation using a fluorescence microscope (LSM 510, Zeiss). The number of integrated cells per eye was determined by counting in serial sections through each eye the number of EGFP positive showing the whole cell body correctly located within the ONL. For each eye, all sections (of 14 μM) were analyzed. Only cell bodies bearing an external segment or/and a synaptic process were included in the counting of mature cells.

Double-labeling analysis was carried out in pictures taken through the microscope with a final magnification of 400× and assessed by visualizing cells expressing the EGFP and another marker labeled in red with the nuclear staining in order to count double-positive cells. To assess the number of double-labeled cells per section, a 400-μm region width, at a distance of 200 μm from the right and the left sides of the optic nerve (ON) region, was considered for counting. At least three slides per eye were analyzed and at least three animals (three retinas) were included per group.

### Post-mortem Human Retina Collection

The adult human retinas analyzed were received from the Eye Bank of Jules-Gonin Eye hospital, and they had been considered to be unsuitable for corneal transplantation. Human retinas were dissected from the rest of the eyeball and frozen at least one night in TRIzol (Invitrogen-Thermo Fisher Scientific, 15596-026) at −80°C. The use of adult human samples for research purposes was supported by the local ethical committee (University of Lausanne, protocol authorization 340/15), and fetal eyes of 12–15 weeks gestation were collected from terminated pregnancies after the receipt of informed, signed, parental consent, according to guidelines approved by the Ethics Committee of Hôpital Ste. Justine. Subjects with ocular malformations were excluded.

### RNA Isolation and Reverse Transcription

RNA was extracted from adult human retinas, according to the manufacturer’s instructions, using TRIzol reagent (Invitrogen-Thermo Fisher Scientific, 15596-026). Briefly, samples were homogenized using 0.2 mL chloroform/1 mL TRIzol reagent, vortexed vigorously, incubated on ice, and centrifuged for a clear phase separation (12,000 rpm, 15 min, 4°C). RNA was precipitated from the aqueous phase by mixing with isopropyl alcohol (0.5 mL/ml TRIzol). After centrifugation (12,000 rpm, 10 min, 4°C), pellets were resuspended in 10–30 μl nuclease-free water (PAA Laboratories). During reverse transcription, to denature any secondary structures in the RNA template, 1 μg total RNA and 1 μl random hexamer primers (Promega, 0.5 mg/ml) were incubated at 70°C for 10 min and immediately cooled on ice. For the reverse transcription reaction, 4 μl AMV-RT buffer (Promega), 2 μl dNTP mix (Promega, 10 mM), 1 μl RNase Inhibitor (RNAsin, Promega), and 1 μl AMV reverse transcriptase (AMV-RT, Promega) were added to a 20 μl final volume reaction and incubated at 42°C for 1 hr, followed by a 10-min incubation at 95°C.

### PCR

Primers were designed using the Primer 3 (http://simgene.com/index.jsp) software, spanning an exon-exon junction where applicable (see Table S1 for mouse qPCR primer list). The reverse and forward primers were used in a multiplex PCR with Taq DNA Polymerase (Invitrogen-Thermo Fisher Scientific) with the following touch down protocol: 5 min at 95°C; one cycle of 30 s at 95°C, 30 s at 60°C, and 30 s at 72°C; one cycle of 30 s at 95°C, 30 s at 58°C, and 30 s at 72°C; and then 29 cycles of 30 s at 95°C, 30 s at 55°C, 30 s at 72°C, and 10 min at 72°C at the end of the run. The reaction mixtures were set up according to the manufacturer’s recommendations (Invitrogen-Thermo Fisher Scientific). Primers were synthesized by Microsynth. Amplification products were resolved on a 1.5% agarose gel. All PCR experiments were performed at least three times on at least three independent human cell samples. Representative images of RT-PCR product are shown of reproducible findings.

The primers used for human samples are as follows:

hCHRNB4 forward, 5′- GAAGACCAGAGTGTCGTTGAG -3′
hCHRNB4 reverse, 5′- ATCCTTGCCTGTTCCACGG -3′
hGAPDH forward, 5′- CGT GTC AGT GGT GGA CCT GAC C -3′
hGAPDH reverse, 5′- CAG GGA CTC CCC AGC AGT GAG G -3′.

### Statistics

Statistical analyses are based on triplicates of at least three independent experiments. All means are presented with ±SEM. Statistical significance was assessed using Graphpad Prism 5 software and applying one-way ANOVA with Tukey’s correction. In figures, *p < 0.05, **p < 0.01, and ***p < 0.001.

## Author Contributions

S.D. performed experimental works and data analyses, contributed to writing, and designed experiments; C.M., F.S., S.C., and A.M. performed experimental work; M.B. provided transgenic mice; M.S. provided transgenic mice and contributed to writing; F.B.-C. wrote to the local ethical committee for the authorization to use human sample for research studies;

Y.A.: designed experiments, data analyses, wrote the paper

## Supplemental Information

Document S1. Figures S1–S8 and Table S1

Document S2. Article plus Supplemental Information
