# Supplementary material for: Cone Genesis Tracing by the Chrnb4-EGFP Mouse Line: Evidences of Cellular Material Fusion after Cone Precursor Transplantation
Source: Mol Ther. 2017 Jan 28;25(3):634–53. doi: 10.1016/j.ymthe.2016.12.015 (PMC5363218; doi:10.1016/j.ymthe.2016.12.015)
Supplement: Document S1. Figures S1–S8 and Table S1 [file mmc1.pdf]

YMTHE, Volume *25*

Supplemental Information

Cone Genesis Tracing by the *Chrnb4*-EGFP Mouse Line: Evidences of Cellular Material Fusion after Cone Precursor Transplantation

Sarah Decembrini, Catherine Martin, Florian Sennlaub, Sylvain Chemtob, Martin Biel, Marijana Samardzija, Alexandre Moulin, Francine Behar-Cohen, and Yvan Arsenijevic

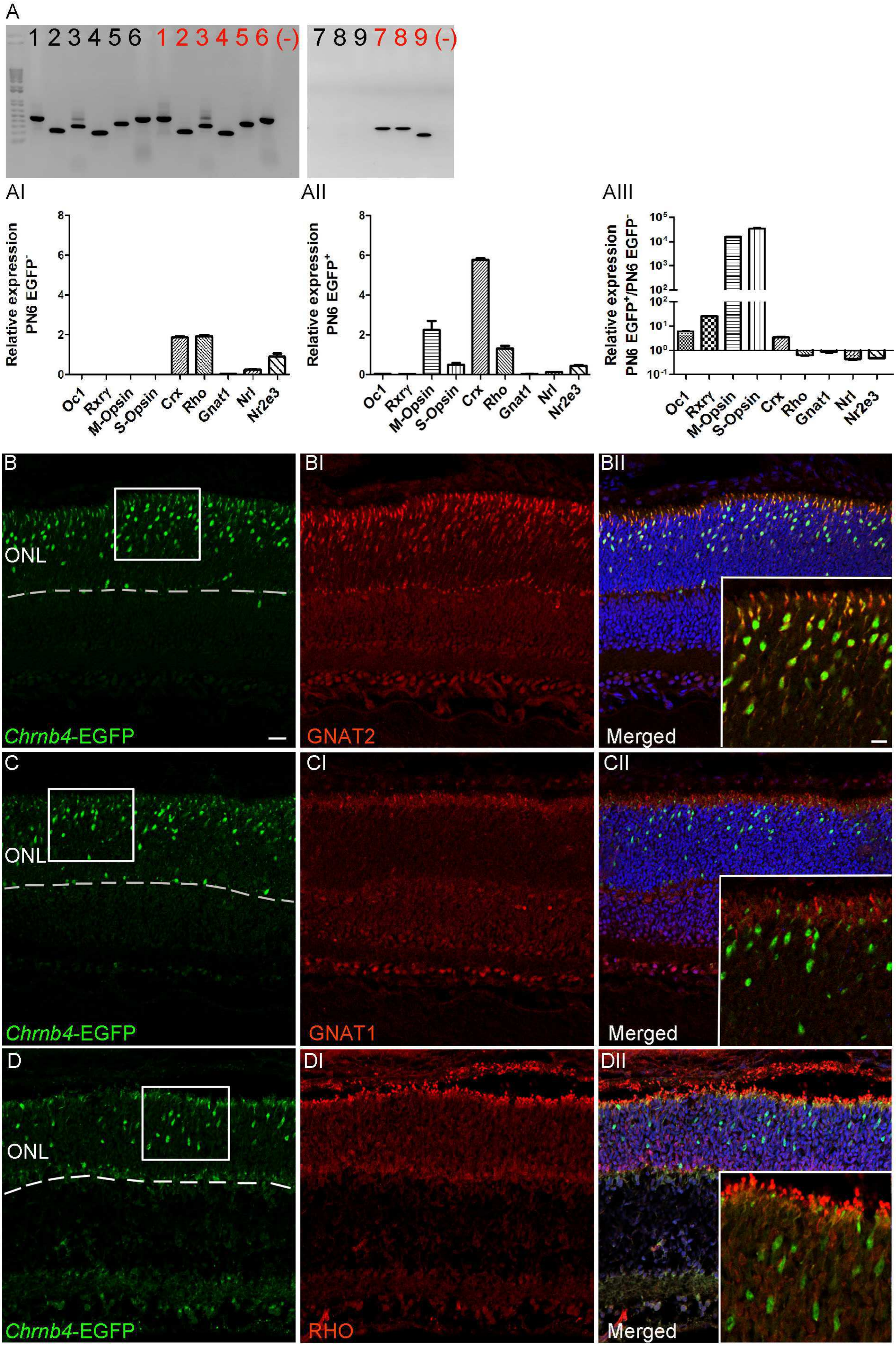
A
1 2 3 4 5 6 1 2 3 4 5 6 (-) 7 8 9 7 8 9 (-)
AI
Relative expression PN6 EGFP⁻
AII
Relative expression PN6 EGFP⁺
AIII
Relative expression PN6 EGFP⁺/PN6 EGFP⁻
Oc1
Rxrγ
M-Opsin
S-Opsin
Crx
Rho
Gnat1
Nrl
Nr2e3
B
ONL
Chrnb4-EGFP
BI
GNAT2
BII
Merged
C
ONL
Chrnb4-EGFP
CI
GNAT1
CII
Merged
D
ONL
Chrnb4-EGFP
DI
RHO
DII
Merged

Supplementary Figure 1. The PN6 *Chrnb4*-EGFP-positive cells show characteristics of newly formed cones. (A) RT-PCR on EGFP-positive (red numbers) and negative (black numbers) cells selected at PN6 isolated by FACS. 1.*Gapdh,* 2.*Gnat1,* 3.*Nr2e3,* 4. *Nrl,* 5.*Crx,* 6. *Rhodopsin*, 7. *Gnat2*, 8. *S-Opsin*, 9. *M-Opsin*. **(AI-III)** Gene expression profile (RT-qPCR) of FACS-sorted PN6 EGFP-positive and negative cells. Normalization performed with Erl8 gene. Photoreceptor specific markers: cone and horizontal precursors (*Oc1*), cone cells (Rxrγ, *Trb2*, *M-Opsin*, *S-Opsin*), photoreceptors (*Crx*) and rod cells (*Rhodopsin, Gnat1, Nrl, Nr2e3*), (n=3). **(B to DII)** PN6 retina sections from the *Chrnb4*-EGFP mouse line. **(B-D)** EGFP is mainly detected in the ONL with cells appearing to migrate to the outer part of the ONL. These EGFP-positive cells express the cone marker GNAT2 **(BI-II)** but not the rod marker GNAT1 **(CI-II)** or Rhodopsin **(D-II).** Inserts in **BII** to **DII** are the enlargements of white squares from **B** to **D**. Abbreviation: ONL: outer nuclear layer. n = 3 analysed sections. Scale bars = 20 µm in A-DII, and B-BIII, 10 µm in inserts.

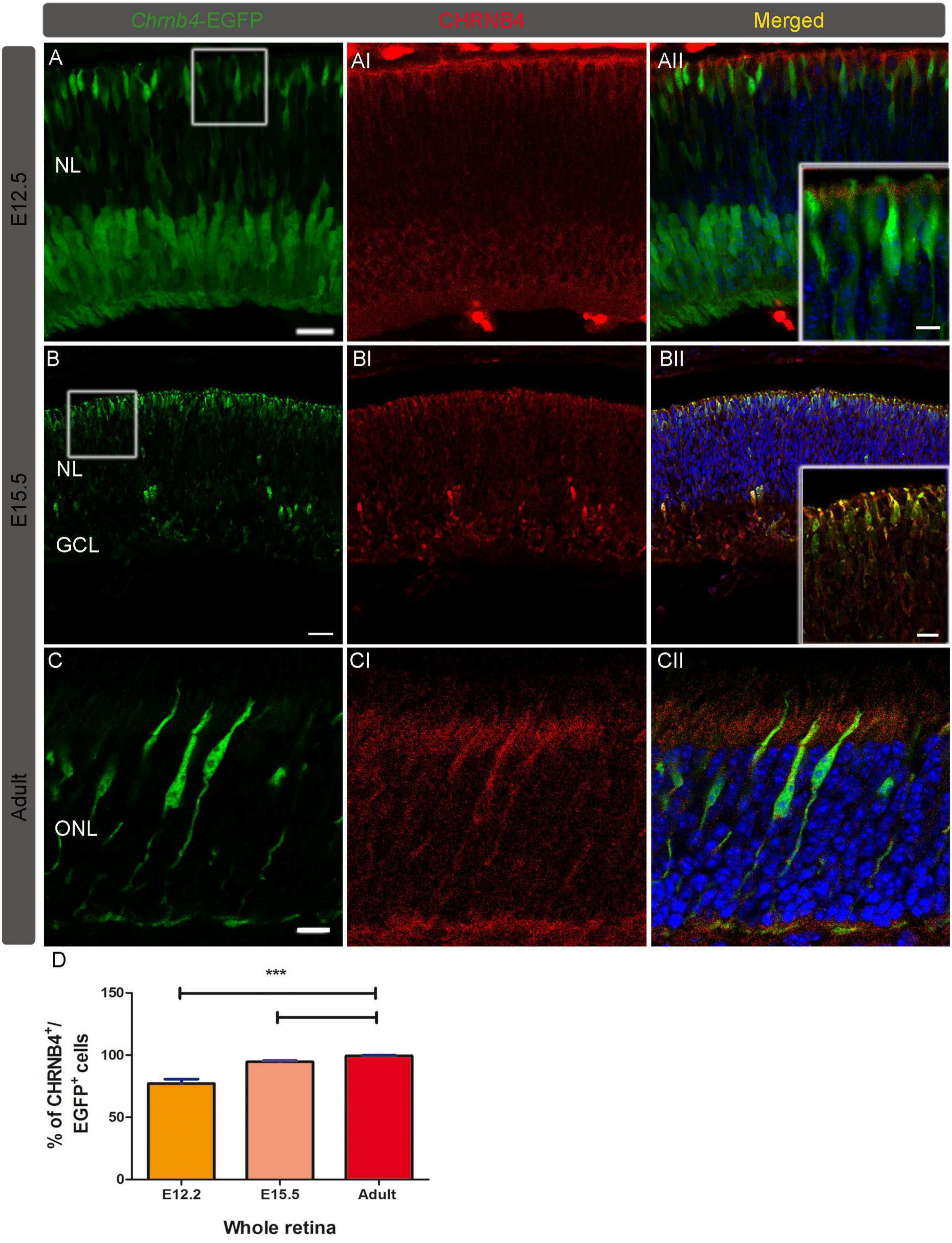

Chrnb4-EGFP
CHRNB4
Merged
E12.5
E15.5
Adult
A
AI
AII
NL
B
BI
BII
GCL
C
CI
CII
ONL
D
***
% of CHRNB4+/ EGFP+ cells
150
100
50
0
E12.2
E15.5
Adult
Whole retina

Supplementary Figure 2. The CHRNB4 protein in the retina is highly co-expressed with the reporter protein of the Chrnb4-EGFP mouse. Transversial sections of E12.5 **(A-AII)** E15.5 **(B-BII)** and adult retina **(C-CII)**. **(A-AII)** CHRNB4 (in red) is faintly expressed in the basal and apical region of the E12.5 retinas. The protein is much more present at E15.5 **(B-BII)** and is then restricted to the cones only in the adult retina **(C-CII).** Inserts in **(AII-BII)** are high magnification micrographs from white squares in **A** and **B**. **(D)** Percentage of CHRNB4-positive cells over all EGFP-positive cells at the analysed ages. ANOVA with Tukey's correction ***P<0.001 (n=3, 5, and 3 per E12.5, E15.5 and adult retinas). Error bars correspond to S.E.M. ONL: outer nuclear layer, NL: Neuroblastic layer, GCL=Ganglion cell layer. DAPI: nuclear staining (blue). Scale bars= 15 µm in A-AII, 30 µm in B-BII and 10 µm in C-CII.

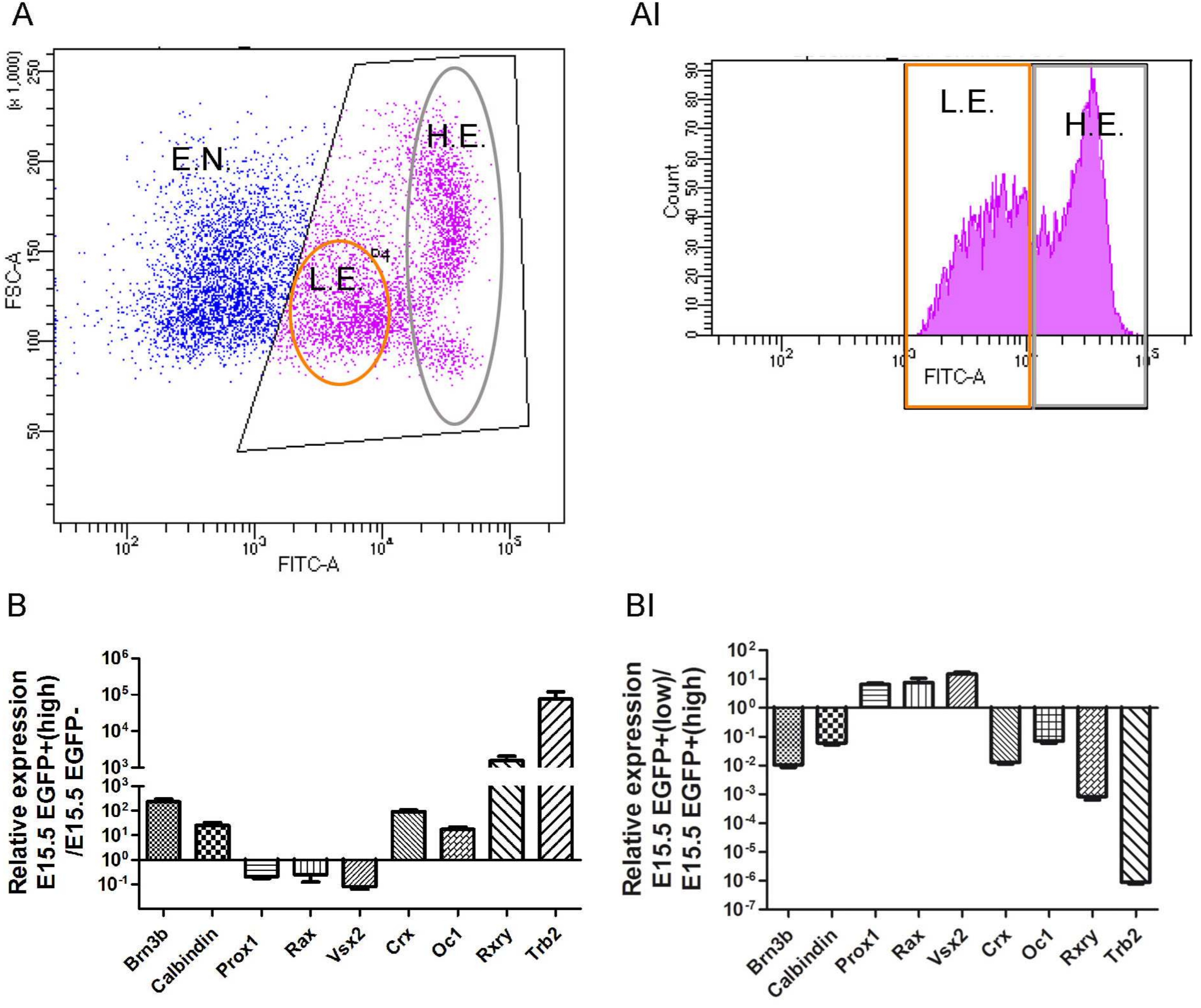
A
AI
E.N.
H.E.
L.E.
P4
FSC-A
(x 1,000)
FITC-A
Count
B
BI
Relative expression
E15.5 EGFP+(high)
/E15.5 EGFP-
E15.5 EGFP+(low)/
E15.5 EGFP+(high)
Brn3b
Calbindin
Prox1
Rax
Vsx2
Crx
Oc1
Rxrγ
Trb2

Supplementary Figure 3. Characterization of E15.5 Chrnb4-EGFP-positive cells isolated by FACS. (A-AI) FACS analysis of E15.5 isolated cells revealed low (LE) and high (HE) EGFP-expressing populations. **(B)** Q-PCR of the high-EGFP population indicates the presence of cone precursor cells (*Crx, Oc1, Rxr□ and Trb2*) as well as cells committed to the RGC and Horizontal cell fates (*Brn3b* and *Calbindin* respectively). **(BI)** Cells expressing a low level of EGFP express genes of the retinal progenitor cells (*Prox1*, *Rax* and *Vsx2*).

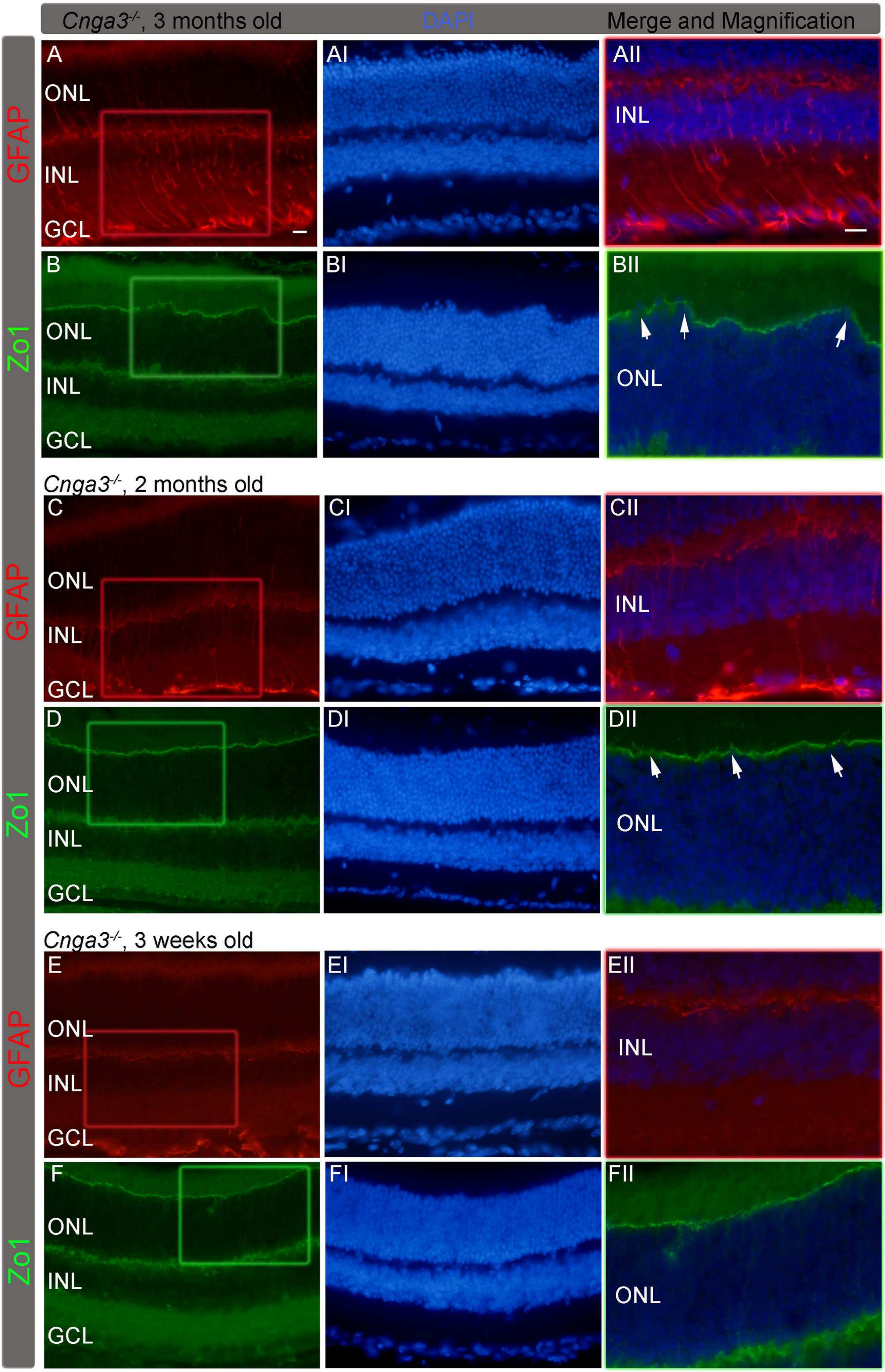

Cnga3-/-, 3 months old
DAPI
Merge and Magnification
GFAP
Zo1
A
ONL
INL
GCL
AI
AII
INL
B
ONL
INL
GCL
BI
BII
ONL
Cnga3-/-, 2 months old
C
ONL
INL
GCL
CI
CII
INL
D
ONL
INL
GCL
DI
DII
ONL
Cnga3-/-, 3 weeks old
E
ONL
INL
GCL
EI
EII
INL
F
ONL
INL
GCL
FI
FII
ONL

Supplementary Figure 4. Glial scarring and outer limiting membrane integrity over time in *Cnga3*$^{-/-}$ mice. (A,AII, C,CII, E,EII) Gliotic response detected by GFAP immunostaining (red) and **(B,BII, D,DII, F,FII)** OLM integrity, evaluated by ZO1 immunostaining (green) in mice at different ages. **(BII, DII)** Rupture of the OLM indicated with white arrows. **(AII-FII)** High magnification of red square in A-F. ONL and INL; outer and inner nuclear layer, GCL, ganglion cell layer. DAPI: nuclear staining (blue). Scale bars = 15 µm in A-F and AI-FI, and 12 µm in AII-FII.

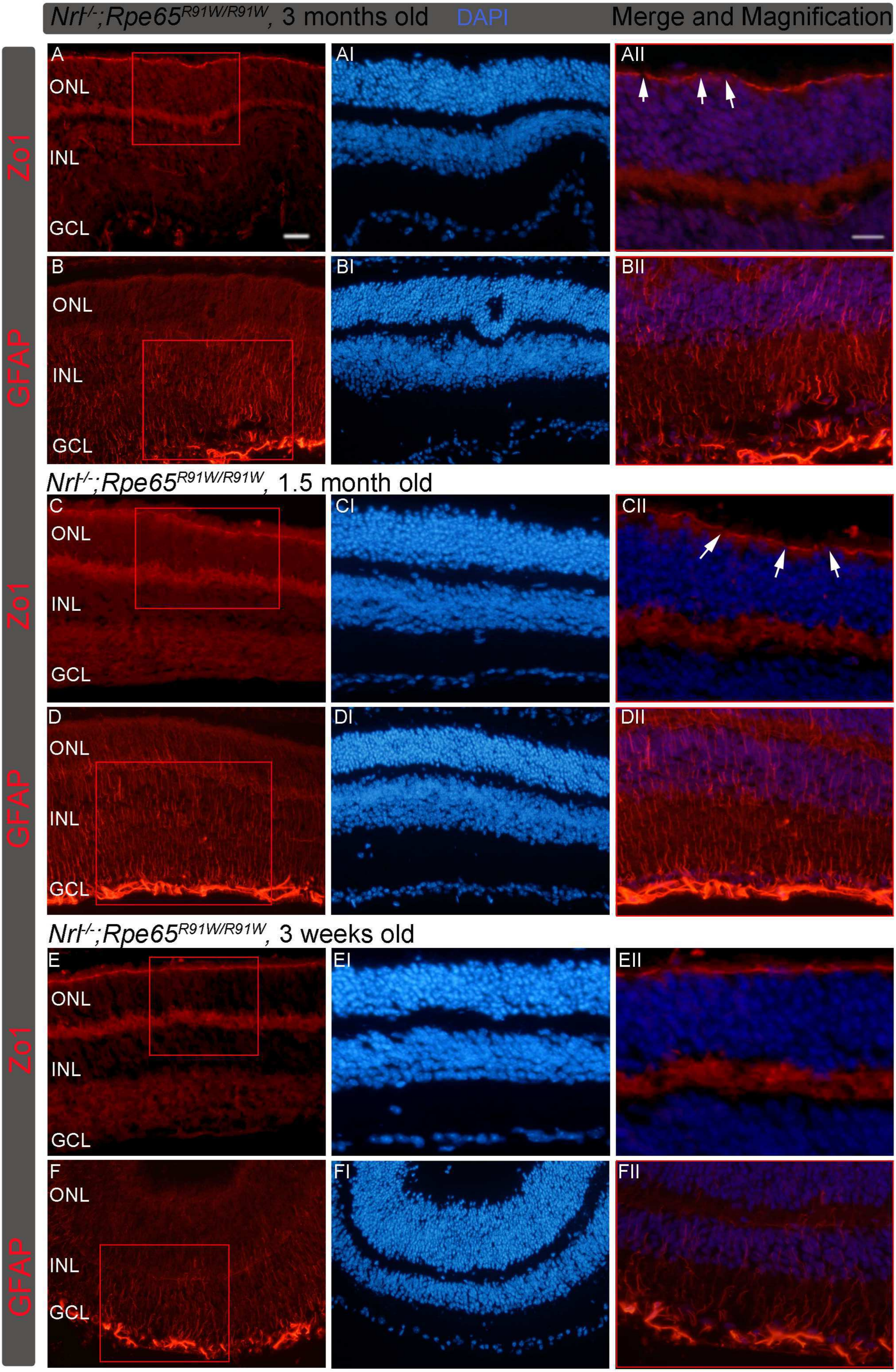
Nrl-/-;Rpe65R91W/R91W, 3 months old
DAPI
Merge and Magnification
A
AI
AII
ONL
INL
GCL
Zo1
B
BI
BII
GFAP
Nrl-/-;Rpe65R91W/R91W, 1.5 month old
C
CI
CII
D
DI
DII
Nrl-/-;Rpe65R91W/R91W, 3 weeks old
E
EI
EII
F
FI
FII

Supplementary Figure 5. Glial scarring and outer limiting membrane integrity over time in *Nrl*$^{-/-}$*;Rpe65*$^{R91W/R91W}$ mice. (B, BII, D, DII, F, FII)) Gliotic response detected by GFAP immunostaining (red) and **(A, AII, C, CII, E, EII)** OLM integrity, evaluated by ZO1 immunostaining (red) in mice at different ages. **(AII-FII)** High magnification of red squares in A-F. Abbreviation: ONL and INL, outer and inner nuclear layer, GCL, ganglion cell layer. DAPI nuclear staining (blue). Scale bars= 10µm in A-AI, B-BI, C-CI, D-DI, E-EI, F-FI and 20µm in AII,FII.

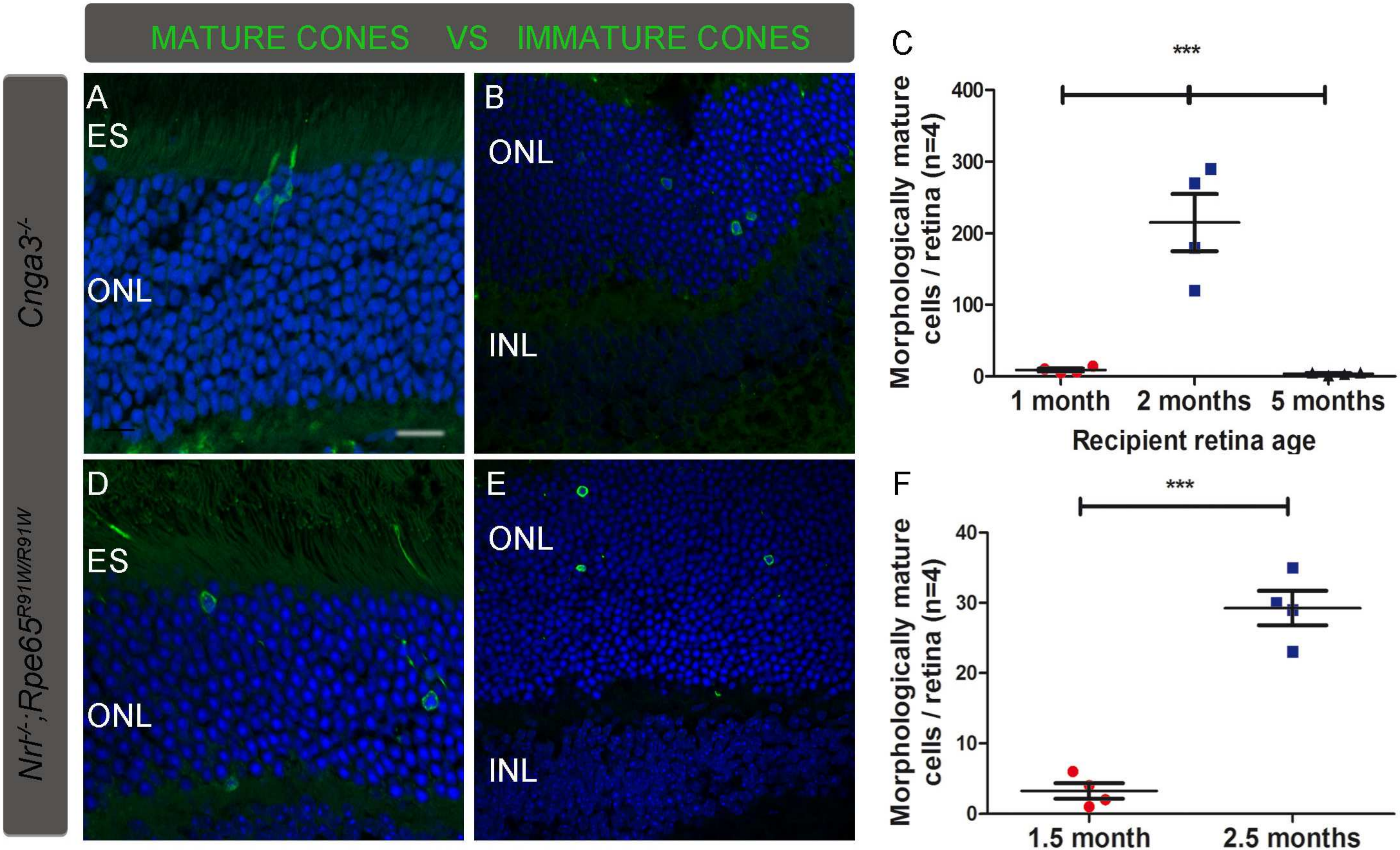
MATURE CONES VS IMMATURE CONES
Cnga3-/-
Nrl-/-;Rpe65R91W/R91W
A
ES
ONL
B
ONL
INL
C
***
Morphologically mature cells / retina (n=4)
400
300
200
100
0
1 month
2 months
5 months
Recipient retina age
D
ES
ONL
E
ONL
INL
F
***
Morphologically mature cells / retina (n=4)
40
30
20
10
0
1.5 month
2.5 months
Recipient retina age

Supplementary Figure 6. A cone dystrophic retina does not favour E15.5 *Chrnb4*-EGFP cell integration after transplantation into *Cnga3*$^{-/-}$ and *Nrl*$^{-/-}$*;Rpe65*$^{R91W/R91W}$ adult retinas. **(A-D)** Examples of mature and **(B, E)** immature cone photoreceptors after transplantation into different recipient retinas. **(C, F)** Quantification of morphologically mature donor cells in the adult retinas defined by the presence of the external segment. Error bars correspond to S.E.M. Abbreviations: ES=External segment, ONL and INL = outer and inner nuclear layer. DAPI nuclear staining (blue) **(D, G, J).** ANOVA with Tukey's correction ***$P<0.001$; n=3. Scale bars=20µm in A, D and 40µm in B, E.

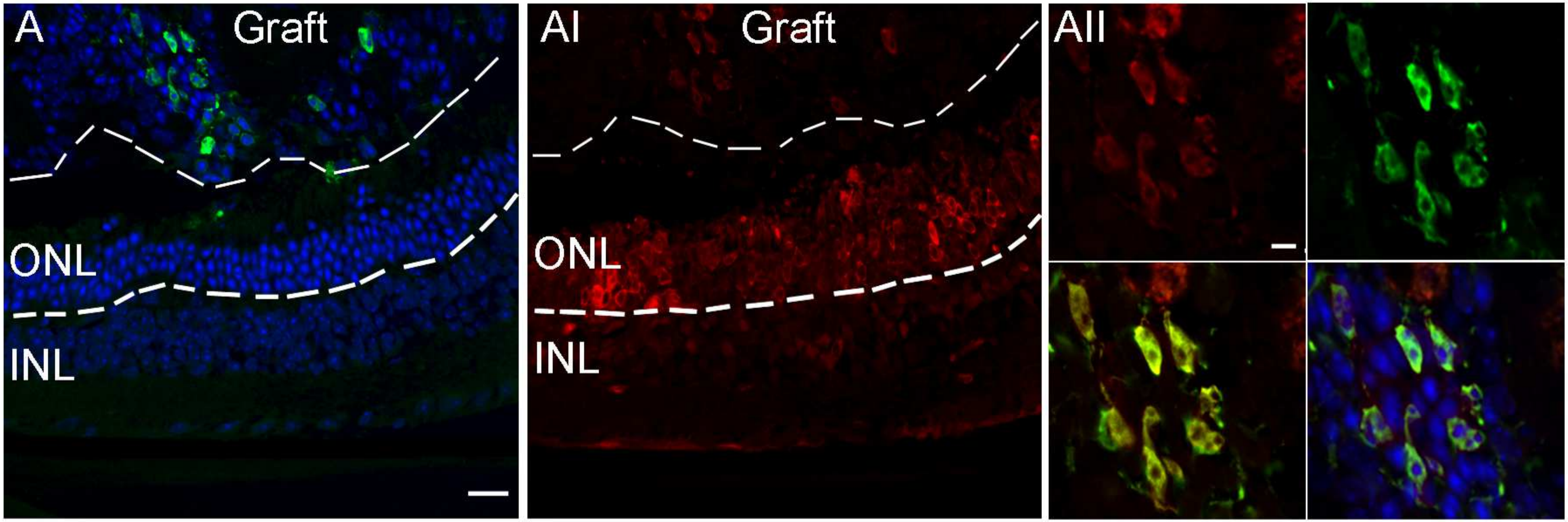
A
Graft
ONL
INL
AI
Graft
ONL
INL
AII

Supplementary Figure 7. Evidence of material exchanges between the host retina towards the graft. (A-AI) Slices of a DsRed retina transplanted with E15.5Chrnb4-EGFP cells. Note the mass of the graft below the retina (blue and green cells above the thin dashed line). (AII) High magnification of EGFP-positive cells in the graft reveal the presence of cells also positive for DsRed. Abbreviations: ONL and INL: outer and inner nuclear layer. Scale bars: 20 µm in A-AI and 5 µm in AII.

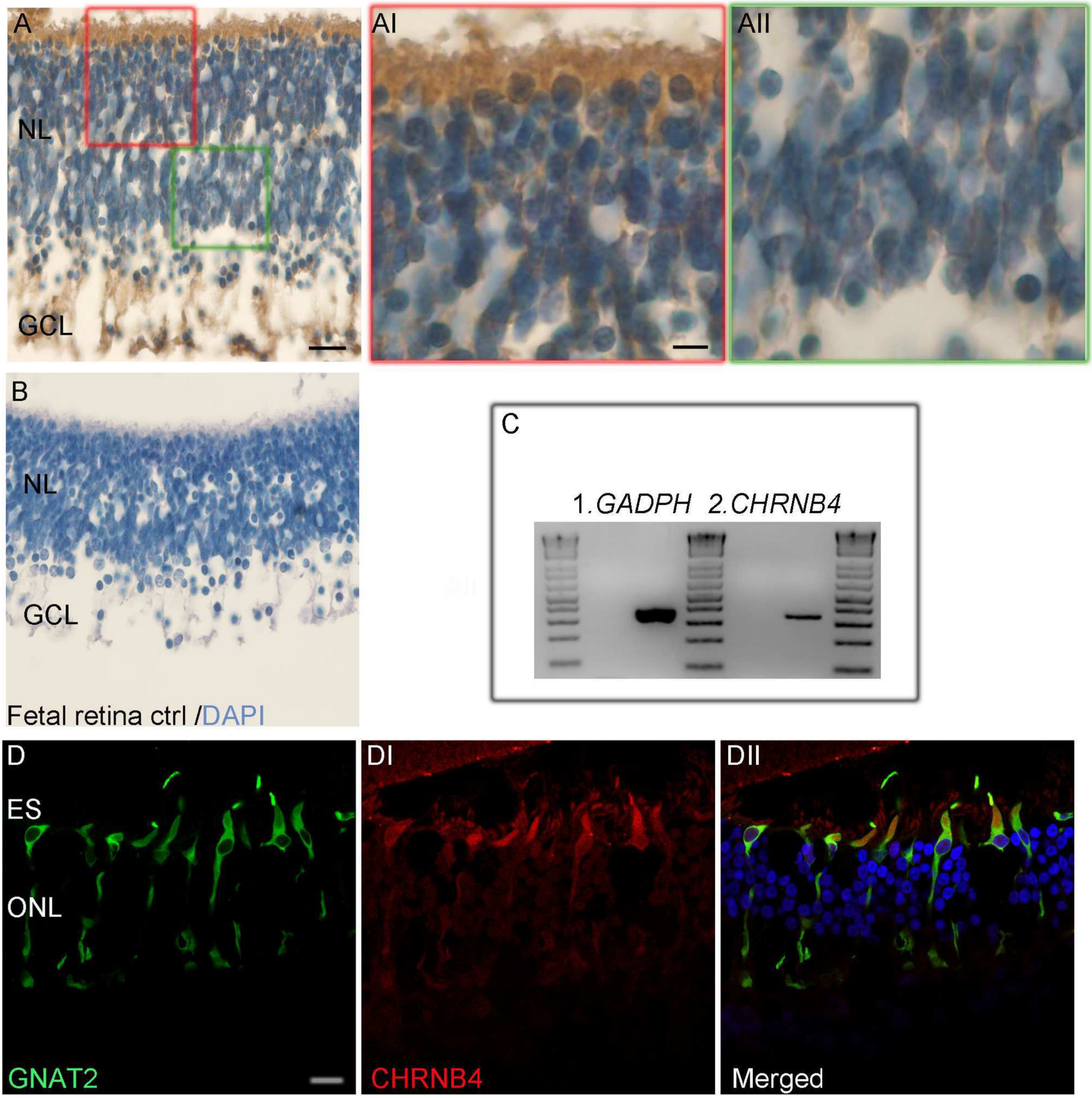
A
NL
GCL
AI
AII
B
NL
GCL
Fetal retina ctrl /DAPI
C
1. GADPH 2. CHRNB4
D
ES
ONL
GNAT2
DI
CHRNB4
DII
Merged

Supplementary Figure 8. CHRNB4 is expressed in human foetal retina and adult cones. **(A-B)** Paraffin section of 3 month old foetal retina. The CHRNB4 protein is expressed in cells close to the apical and basal retinal side (dark brown). **(AI, AII)** Apical and basal magnification of (A) respectively. Note the staining in the cell periphery compatible with the localization of a receptor in the cell membrane **(B)** Staining of foetal retina without the first antibody (control, ctrl). **(C)** RT-PCR of *GAPDH* and *CHRNB4* from retina of a 78 year old donor. **(D-DII)** Double staining of GNAT2 (in green) and CHRNB4 (in red) in an adult retina from the same retina donor. ONL: outer nuclear layer, NL: neuroblastic layer, GCL: ganglion cell layer, ES: external segment. DAPI: nuclear staining (blue). Scale bars = 20 µm in AI-AII, C-CII and 50µm in A, B.

Primer list for qPCR

mGnat2

FW gagggagtcacctgcatcat
RV aagactcgtgcatgcgatt

mRxry

FW tcctccaggaatcaacttgg
RV agctgctgacactgttgacc

mCrx

FW ccccaatgtggacctgat
RV ggctcctggtgaatgtggt

mGnat1

FW agagctggagaagaagctgaaa
RV tagtgctcttcccggattca

mProx1
FW cgacatctcaccttattcagga
RV ttgccttttcaagtgattgg

mNrl
FW ttctggttctgacagtgactacg
RV tgggactgagcagagagagg

mOnecut1
FW agaccttccggaggatgtg
RV ttgctctttccgtttgcag

mVsx2
FW cgtaagaagcggcgacac
RV tctgggtagtgggcttcatt

mGapdh
FW gggttcctataaatacggactgc
RV ccattttgtctacgggacga

mBrnb3
FW gcgctacacagtgcctcccc
RV actctcatccagcccgccga

mRhodopsin
FW acctggatcatggcgttg
RV tgccctcagggatgtacc

mNr2e3
FW atctatgcctgcaatggctg
RV tctggttgcagacacagacg

mTrb2
FW gcactgaagaatgagcagac
RV acacagagctcatctttgtc

mCalbindin
FW ctcaaactagccgctgcac
RV gctcctggatcaagttctgc

mErl8
FW actggacagttcgtgtacth
RV gcttcactcgagtcttcttg
